# Exploring the perspectives of key stakeholders on the design and delivery of a cognitive rehabilitation intervention for people post-stroke

**DOI:** 10.1371/journal.pone.0269961

**Published:** 2022-06-16

**Authors:** Mairéad O’ Donoghue, Pauline Boland, Siobhan Leahy, Rose Galvin, John McManus, Dominika Lisiecka, Sara Hayes

**Affiliations:** 1 School of Allied Health, Ageing Research Centre, Faculty of Education and Health Sciences, Health Research Institute, University of Limerick, Limerick, Ireland; 2 Department of Sport, Exercise & Nutrition, Atlantic Technological University (ATU), Galway, Ireland; 3 Consultant in Geriatric and Stroke Medicine, University Hospital Limerick, Limerick City, Ireland; 4 Department of Nursing and Healthcare Sciences, School of Health and Social Sciences, Munster Technological University Kerry Campus, Tralee, Kerry, Ireland; Flinders University, AUSTRALIA

## Abstract

**Purpose:**

Stroke is a leading cause of death and disability worldwide. Despite the prevalence and associated burden of post-stroke cognitive impairment, there is uncertainty regarding optimum interventions to improve cognitive function in people post-stroke. The aim of this study is to explore the perspectives of key stakeholders on the design and development of a multidisciplinary intervention to rehabilitate cognitive deficits in people post-stroke.

**Materials and methods:**

Audio-recorded, semi-structured interviews were employed with people post-stroke, caregivers, healthcare professionals and academics. All transcribed interviews were exported to NVivo software and analysed using reflexive thematic analysis.

**Results:**

Thirty interviews were conducted across stakeholder groups including people post-stroke (n = 10), caregivers (n = 5), healthcare professionals (n = 14) and academics (n = 1). Four themes relevant to the design and development of the intervention were identified (i) engagement in the intervention must be meaningful, (ii) the point of readiness to engage, (iii) a familiar but flexible setting is key (iv) pragmatics of intervention delivery.

**Conclusions:**

These findings present new perspectives across stakeholder groups on the design and delivery of an intervention to rehabilitate cognitive deficits in people post-stroke. Taken together with existing quantitative evidence, these findings will inform the development of a feasibility trial, examining patient and process outcomes, to rehabilitate cognitive deficits post-stroke.

## Introduction

Stroke is among the leading causes of death and disability adjusted life years worldwide [1]. The incidence of cognitive impairment in people post-stroke (PpS) varies from 20% to 80% depending on the aetiology of stroke [2–4]. Over 50% of PpS who recover well from physical effects of stroke continue to experience cognitive deficits in the longer term [5]. Cognitive impairment prevalence for people at five years post-stroke has been noted as 22%, reducing to only 21% at 14 years post-stroke, according to a UK based prospective population-based stroke register [6]. Cognitive impairment post-stroke has been shown to be independently associated with a lower quality of life [7], higher rates of mortality and institutionalisation [8], increased caregiver burden [9] and increased healthcare costs [10].

Despite this, much rehabilitation focus is placed on the improvement of physical deficits post-stroke, with less emphasis on cognitive impairments [11, 12]. Peoples et al. [13] reported from their synthesis of qualitative studies that PpS report a focus during rehabilitation on physical needs over non-physical needs such as social re-integration and psychological support. These non-physical needs were perceived as factors that could enable the PpS to regain control over their everyday life [13]. Similarly, McKevitt et al. [14] estimated the prevalence of self-reported unmet needs in community-dwelling stroke survivors (n = 799) across the United Kingdom and found that 60% of those surveyed reported memory problems after stroke as an unmet need. A priority setting partnership in the UK, the James Lind Alliance, identified that cognitive impairment post-stroke was among the top priorities for stroke research [15].

Cognition is not a unitary concept, as evidenced by the variety and breadth of neuropsychological assessments available [16]. Cognitive impairment post-stroke can manifest as a variety of deficits across multiple domains of cognitive function [17] which are important to enable someone to select and process information [18]. Previous Cochrane reviews have explored the effectiveness of cognitive rehabilitation interventions on a specific domain of cognitive function post-stroke, such as attention, memory, executive function, limb apraxia, neglect and perception [19–24]. An overview by Gillespie et al. [25] synthesised evidence across these Cochrane reviews and concluded that while there are some short-term benefits following cognitive rehabilitation on specific cognitive domains, the effectiveness of these interventions has yet to be established. Furthermore, these improvements were not likely to persist in the long-term and did not improve the everyday functioning of the individual post-stroke [25].

Cognitive rehabilitation is defined as “a systematic, functionally oriented service of therapeutic activities that is based on assessment and understanding of the patient’s brain-behavioural deficits” [18]. O’Donoghue et al. [26] conducted a systematic review of 64 studies addressing any type of non-pharmacological rehabilitation intervention which may improve cognitive function in people post-stroke. A range of rehabilitation interventions were identified across the 64 included studies with evidence to support multiple component interventions, physical activity interventions and non-invasive brain stimulation to improve cognitive function post-stroke [26]. The current study builds on the findings of this systematic review by ascertaining the perspectives of stakeholders across a multitude of rehabilitation interventions ranging from multicomponent interventions to physical activity interventions to occupational-based and cognitive rehabilitation interventions.

A previous qualitative enquiry gathered the perspectives of key stakeholders on the design of a cognitive rehabilitation intervention post-stroke, focusing solely on psychological interventions [27]. This previous qualitative study was underpinned by a systematic review of non-randomised controlled studies of psychological interventions [28] and highlighted the need for improving confidence and self-efficacy in the management of cognitive impairment post-stroke, in addition to the importance of effective information provision on stroke sequalae and psychoeducation regarding the consequences of stroke [27]. In contrast to the qualitative enquiry of Merriman et al. [27], the current study elicits perspectives on all types of non-pharmacological rehabilitation interventions, not only psychological interventions, for cognitive impairment post-stroke, as evidenced from our previous systematic review [26].

Living with memory deficits may result in negative effects on the PpS and their family once in the community such as fragmented care, marginalisation and lack of proactive follow-up [29]. A recent systematic review and meta-ethnography found that PpS and carers may feel abandoned by healthcare services due to lack of continuity of care, limitations in access to services and inadequate information and opportunity to re-engage with services [30]. Specifically, people with memory problems post-stroke and their carers have identified additional barriers such as fear of a dementia diagnosis, reduced insight into cognitive deficits and the lack of familiarity with healthcare professionals (HCPs) to comfortably discuss their memory problems [31]. These perceived unmet needs and inequities in accessing rehabilitation services are challenges that require attention. The presence of general cognitive impairment, as measured from the Montreal Cognitive Assessment scale (MoCA) is associated with a lower quality of life at three months post-stroke (p = 0.003) [32]. Moreover, the highest proportion of unmet needs post-stroke fall into the cognitive, communication and anxiety/ depression domains [14, 33]. The unmet needs for cognitive impairment in PpS result in a significantly lower quality of life, as measured by the EQ-5D index, even after adjusting for age, sex, and modified Rankin scale scores [33]. In consideration of the burden of cognitive impairment post-stroke, coupled with cognition being among the top research priorities in stroke [34], the design of an effective and feasible intervention is an urgent issue.

Given that key features of cognitive intervention design remain inconsistently defined [35], it is imperative to explore the insights of key stakeholders regarding their engagement with such interventions and their perceived effectiveness. The engagement of stakeholders is essential to the development of a cohesive stroke system of care [36]. Qualitative research methods are key components in the conduct of research into complex interventions, by increasing knowledge about intervention components and mechanisms of action [37, 38]. The Medical Research Council’s (MRC) guidelines for developing complex interventions details the importance of identifying the current evidence base [39]. Thus, in accordance with the MRC framework for developing and evaluating complex interventions [39], perspectives of relevant stakeholders will be used to inform the development and design of an intervention to rehabilitate cognitive deficits in PpS. Taken together with quantitative evidence from our systematic review [26], the findings from the present study will inform the development of an evidence-based and stakeholder-informed feasibility study to rehabilitate cognitive deficits post-stroke. To this end, this study aims to elicit the perspectives of PpS, caregivers, HCPs on the design and delivery of an intervention to rehabilitate cognitive impairment post-stroke.

## Methods

This study employed a qualitative approach with reflexive thematic analysis of data [40, 41]. The study is reported in line with the Consolidated Criteria for Reporting Qualitative Research (COREQ) checklist [42]. The perspectives of key stakeholders were gathered using a semi-structured interview script via telephone or telecommunication platform (Microsoft Teams). Interview scripts are available as S1 Appendix.

### Sampling and recruitment

A pragmatic, purposive sampling technique using snowball sampling was used in this study [43, 44]. Participants across three stakeholder groups were recruited via their respective gatekeepers: PpS, caregivers, HCPs with an interest in stroke rehabilitation. Information power, as defined by Malterud et al. [45] indicates that the more information the study sample holds that is relevant to the objectives of the study, the lower the number of participants that are needed. Sufficient information power depends on the aim of the study, sample specificity, use of established theory, quality of the interview dialogue and the nature of data analysis [45].

PpS were recruited via a local stroke support group and via a local brain injury service-Headway, in Limerick, Ireland. Invitation letters and participant information leaflets for the study were sent to gatekeepers of both these sites. The invitation letter was sent prior to the participant information leaflet. The letter provided a summary of the study to allow a potential participant to consider their interest. If participants were happy to find out more, a participant information leaflet was provided and participants were given a minimum of one week to consider participation prior to informed consent being sought. During this time, potential participants were encouraged to seek advice from their gatekeeper regarding the study and that any queries would be addressed by MOD, without any pressure to participate in the study. As the optimum timing of cognitive rehabilitation post-stroke remains unclear [35], recruitment of people post-stroke was not specified to either inpatient, rehabilitation or community settings.

PpS were included if they were:

- Aged 18 years or older with a diagnosis of stroke
- Self-reported cognitive problems post-stroke
- Able to verbally communicate over the phone/ via telecommunication platform
- Able to provide informed consent. Ability to provide consent was determined at the eligibility checking stage though the gatekeepers.

Caregivers included spouses or family members who provide care (paid or unpaid), support or assistance to PpS [46]. Caregivers, who were spouses of PpS, were recruited via gatekeepers at a local stroke support group and via a local brain injury service, Headway in Limerick, Ireland. For paid caregivers, invitation letters were sent through a gatekeeper from the Carers Association Limerick.

HCPs were recruited from relevant clinical sites such as stroke units of acute hospitals, rehabilitation units of subacute hospitals or community settings, as well as via social media platform Twitter. HCPs were eligible for inclusion if they had current or previous experience treating people post-stroke with cognitive impairment. HCPs included physiotherapists (PTs), occupational therapists (OTs), psychologists and speech and language therapists (SLTs) working in the provision of stroke rehabilitation services. Academics included those with research interest in stroke rehabilitation.

A week after individuals had received the participant information leaflet, potential participants were contacted by telephone once they expressed an interest in taking part, to ensure they had read and understood the participant information leaflet. MOD confirmed eligibility with each participant and ensured informed consent was recorded. According to the Assisted Decision-Making Capacity Act (2015) [47], individuals should be assumed to have the capacity to consent unless otherwise demonstrated. To this end, the capacity of PpS was determined at eligibility checking by gatekeepers working in rehabilitation settings with people post-stroke. Informed consent was subsequently checked when outlining the participation information leaflet; MOD ensured participants demonstrated an understanding of what participation entailed, any questions regarding participation were answered by MOD, while re-iterating participants’ rights from the initial signing of the consent form, to re-iteration of informed consent on the day of the interview with MOD and finally, once the interview was completed.

Consent forms were discussed with participants to ensure participants’ understanding of the nature of their participation in this study [48]. MOD referred to the INVOLVE guidelines regarding the knowledge, skills and experience required to participate in patient and public involvement (PPI) when addressing participant queries [49]. this study employed strategies such as reducing the cognitive load on individuals by lessening the content of the interview line of questioning and utilising clear, concise communication [50]. Furthermore, people post-stroke were interviewed remotely while situated in their home to provide a familiar and relaxed environment to facilitate open communication during the interview process and in which individuals were more likely to disclose information relating to the nature of their lived experiences [48].

### Data collection

Audio-recorded, semi-structured interviews were conducted via telephone or via telecommunication platform such as Microsoft Teams, as per the participants’ preference [51]. The interview guide, (S1 Appendix), was informed by our previous systematic review [26], background literature and the template for intervention description and replication (TIDieR) checklist [51]. The Template for Intervention Description and Replication (TIDieR) checklist TIDier was used as a tool to ensure a detailed description of specific intervention components, specifically, the who, what, where, when, why and how of the intervention design [51]. The interview script was piloted with one PpS, one caregiver and one HCP to assess the clarity and appropriateness of interview questions. Minor changes were discussed with each person who provided pilot feedback to ensure clarity of understanding. For example, the interview line of questioning for PpS was shortened to allow for concise, clear questions that would reduce the cognitive load on individuals during the interview process. Field notes were documented after all interviews. All interviews were conducted by MOD, a female physiotherapist and PhD student. As part of her postgraduate studies, MOD has completed training in qualitative research which was centred on the development of the skills of early-career researchers in qualitative research methods. MOD was running an exercise class with some of the PpS with whom interviews were conducted, however they were approached about the study by an independent gatekeeper. Interviews continued until information power was reached (45). Participants were made aware that this study would form part of MOD’s doctoral studies. Ethical approval was obtained from the Faculty of Education and Health Sciences Research Ethics Committee in the University of Limerick [Ref 2020_03_05_EHS].

### Data analysis

All audio-recorded interviews were fully transcribed verbatim by MOD. Nvivo software package (Version 12 QSR International) was used to organise and retrieve data. Data were collectively analysed using reflexive thematic analysis (TA) [40]. Reflexive TA was employed in this research due to its theoretical flexibility, acknowledging the researcher’s own perspectives and biases when interpreting the data. For example, given the physiotherapeutic background of MOD, it was reflected upon and discussed in peer debriefing sessions how MOD may be drawn to more physical activity interventions. To this end, reflexive practice was facilitated through continuous peer debriefing between MOD and the research team (SH, PB, DL), along with the completion of fieldnotes and a reflexive diary following each interview (MOD) [52]. In addition, continuous discussion took place between MOD, a novice researcher, and PB, an experienced qualitative researcher, to ensure accuracy and clarity in the interpretation of the dataset. Inter-coder reliability was not assessed in this qualitative inquiry due to a reflexive TA approach being adopted [53]. In line with reflexive TA, data were analysed using an inductive approach, where generated codes were based on the semantic meanings of the primary data rather than existing theories or concepts [53, 54].

The six stages of reflexive TA [40, 55] were followed, in line 20 critical questions to guide the quality of the reflexive TA process to ensure methodological rigour [53]: (i) data familiarisation involved repeated engagement with fieldnotes and transcripts. Memos about initial key patterns were noted in NVivo. Initial theoretical and reflexive thoughts were documented to inform the next step; (ii) generation of initial codes; all transcripts were coded by MOD using an open-coding approach. A purposive set of transcripts were also coded by PB to review initial coding and discuss any disagreements; (iii) conceptualisation of themes involved the identification and interpretative analysis of the collated codes into preliminary themes; (iv) reviewing of themes involved the refinement of themes identified. This required in-depth interpretation and reviewing of the boundaries of each theme and probing to decipher if there was sufficient data to support the theme. This stage was conducted alongside peer debriefing sessions with other co-authors PB, SH and DL. Stage four concluded with clear and identifiable distinctions between themes with supporting data; (v) defining and naming of themes required clear and descriptive working definitions to be generated for each theme and potential subtheme. This resulted in clarifying the scope of each theme and editing the theme titles to reflect a description of the central concept of the theme. At this point, themes were summarised and sent to stakeholder groups via their respective gatekeepers to allow for participant feedback on themes; (vi) Producing the final narrative report involved the writing up of the analysis into the publication of a journal article.

## Results

The concept of information power was followed as a pragmatic guiding principle to estimate an approximate number of participants required for sufficiently rich data for analysis [45]. A stepwise approach was followed from the initial stages of data collection, through to data analysis and peer debriefing sessions. MOD, a novice researcher and physiotherapist, engaged in regular peer debriefing sessions with PB, an experienced qualitative researcher and occupational therapist, where repeated appraisal of information power was supported by preliminary, and then iterative, analysis of data and themes as they were generated and continuously refined. Recruitment was ceased when no new themes were identified and therefore sufficient information power was established, as defined by Malterud et al. [45]. To this end, sufficient information power was deemed to be reached in the current qualitative inquiry at a sample size of 30 participants: PpS (n = 10), caregivers (n = 5), healthcare professionals (n = 14) and academics (n = 1). Over the course of recruitment, three potential participants declined to interview including one PpS and two caregivers. Please see a summary of participant demographics outlined in Table 1 (PpS), Table 2 (Caregivers) and Table 3 (HCPs).

**Table 1:** Participant demographics: People post-stroke (n = 10).

ID	Age	Gender	Time since stroke	Stroke subtype	Interview length	Occupation
PpS_1	67	Male	6 years	Right sided Ischemic	22:15	Unemployed since stroke
PpS_2	69	Male	15 years	Right sided Ischemic	24:11	Unemployed since stroke
PpS_3	72	Female	8 years	Haemorrhagic	26:22	Unemployed since stroke
PpS_4	46	Female	5 years	Right sided Ischemic	21:35	Part time employment
PpS_5	21	Female	3 years	Haemorrhagic	27:35	Student-attends university
PpS_6	60	Male	7 years	Haemorrhagic	22:47	Unemployed since stroke
PpS_7	61	Female	4 years	Left sided Ischemic	20:52	Unemployed since stroke
PpS_8	67	Female	5 years	“Not sure”	18:22	Unemployed since stroke
PpS_9	68	Female	17 years	Ischemic	35:05	Unemployed since stroke
PpS_10	71	Male	3 years	Left sided Ischemic	21:16	Retired plumber

**Table 2:** Participant demographics: Caregivers (n = 5).

ID	Age	Gender	Relationship	Employment	Interview length
Carer_1	61	Female	Wife	Teacher	53:25
Carer_2	65	Female	Wife	Retired	32:35
Carer_3	67	Female	Wife	Business owner	35:54
Carer_4	62	Female	Wife	Retired	29:34
Carer_5	61	Female	Wife	Part time farmer	27:16

**Table 3:** Participant demographics: Healthcare professionals (n = 15); Physiotherapists (PTs) (n = 6), Occupational therapists (OTs) (n = 6), Speech and Language therapists (SLTs) (n = 2) and academicians (n = 1).

ID	Age	Gender	Setting	Time in this role	Interview length
OT_1	47	Female	Inpatient acute	1 year	34:17
OT_2	25	Female	Inpatient acute	1 year	28:15
OT_3	36	Female	Inpatient acute and ESD	Over 10 years	51:52
OT_4	48	Female	Inpatient acute	6 years	36:10
OT_5	41	Female	Inpatient acute	3 years	33:15
OT_6	39	Female	Outpatient and community	13 years	34:01
PT_1	28	Female	Community	2 years	33:17
PT_2	39	Female	Acute stroke unit	12 years	28:09
PT_3	40	Female	Inpatient acute	11 years	43:33
PT_4	48	Male	Inpatient and community	Over 20 years	37:23
PT_5	46	Female	Inpatient acute	12 years	33:42
PT_6	38	Female	Inpatient acute	14 years	28:17
SLT_1	38	Female	University	1 year	56:38
SLT_2	27	Female	Inpatient acute	3 years	37:30
SLT_3	37	Female	Outpatient	13 years	36:05

**Fig 1:**
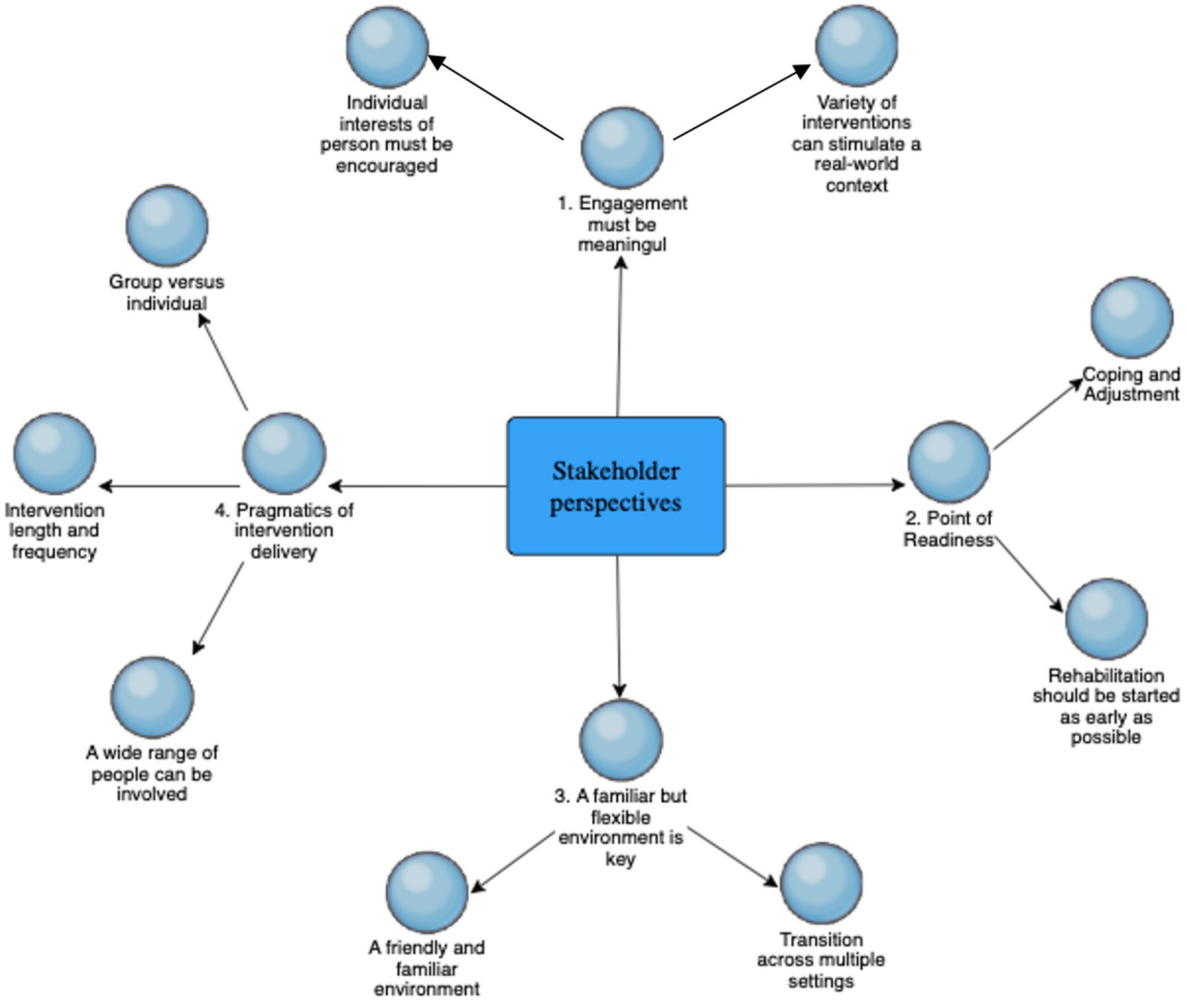
Concept map of key themes and subthemes.

Our findings are outlined through four key components of intervention design, namely (i) engagement must be meaningful, (ii) the point of readiness to intervene (iii) a familiar, but flexible setting is key and (iv) pragmatics of intervention delivery. Fig 1 provides a visual representation of these four main themes, and associated sub-themes, which each discussed in turn below.

### Engagement must be meaningful

#### Individual interests of PpS need to be encouraged

The majority of participants across each stakeholder group described the importance of engaging PpS in accordance with their own interests and passions in life: *“Music*, *I used to love music*, *singing and that type of thing*. *And acting*, *acting in a play or anything like that*, *I found fantastic*. *They were great for memory because you had to memorise an awful lot of stuff”* [PpS_ 2]. Where PpS had a prior interest in an activity, their participation in the activity was higher. HCPs encouraged this type of intervention delivery wherein the PpS could understand the relevance of tasks, as opposed to performing brain training exercises such as crosswords or puzzles without giving context to these tasks. Meaningful participation was described by both carers and HCPs as a means to reduce the cognitive load of tasks, as the novelty of intervention was thereby less of a challenge: *“I do them [pen and paper tasks] but only if the patient can grasp the relevance and the meaningfulness… They have to know why it’s relevant and why it’s important and what it’s working on in real life tasks”* [OT_3].

HCPs discussed how it could be challenging to transfer gains observed during formal cognitive rehabilitation to the everyday life of the PpS: *“I think*, *there’s possibly an issue around… the sort of ecological validity of assessments and results*. *So if you perform well on a pen and paper task*, *to what extent does that improvement carry over to everyday life*? *And so I imagine that*, *yeah*, *you improve the task but there’s a wider question of how does that help the person*?*”* [SLT_1]. Both caregivers and HCPs described the importance of a real-world context to intervention: *“I suppose again*, *the meaningfulness*, *the timing*, *the environment*, *the goal of [the intervention] would have to be very explicit”* [OT_3]. Context-specific, meaningful goals were noted to promote engagement, “*If somebody wants to be able to return to do the shopping*, *it would involve actually walking out to the shop*, *making it as real-world as possible you know*, *and we bring physical activity into it as much as possible”* [OT_4].

#### Variety of interventions can stimulate a real-world context

Several types of interventions were perceived as valuable, one of which was a multicomponent approach to cognitive rehabilitation post-stroke. The description of multicomponent interventions most commonly involved a form of cognitive training and psychoeducation, in conjunction with a form of physical activity. Again, the importance of meaningful intervention with transference of effects to the everyday life of the PpS was iterated by both caregivers and HCPs: “*I imagine the benefits of having multiple components is that you might tap into the sort of outcome that you want somebody to have in their everyday life a little bit more*, *you might be kind of making the task a bit harder or more sort of ecologically valid*, *that you might have to kind of think on your feet while you’re doing another thing you know”* [SLT_1]. PpS and caregivers sometimes used the word “holistic” when referring to interventions which incorporated multiple components: *“Yeah*, *you see*, *I obviously would think the combination is great*, *because I think a holistic approach is the way to go… I’m a huge believer in the physical*, *spiritual*, *emotional*, *social*, *yeah”* [Carer_1].

Occupational interventions, through which PpS were helped to modify tasks in order to carry out activities of daily living were seen as beneficial. These included strategies for remembering weekly tasks, organising of the schedule of the PpS and re-learning everyday tasks such as dressing and cooking. One PpS described the strategies taught to them by their occupational therapist when relearning to shower independently: *“Well I struggled with showering… How do I explain this now*, *they had a mirror and you had to try and reach forward and move your hand in a circle*, *to teach you how to move the affected hand”* [PpS_3]. One physiotherapist referred to these types of occupational-based interventions as the “cornerstone” of rehabilitating cognitive deficits post-stroke and alluded to the benefits of an interdisciplinary approach in delivering this type of intervention. Again, the meaningfulness of the included activities was emphasized: *“I mean*, *are you only thinking of one person doing this*? *Because most appropriate would be both disciplines bringing in their experience together…I think that is the cornerstone of it to be honest*, *if I was retraining gait*, *I would be sweeping the floor or stacking a dishwasher*. *That’s key*, *doing joint sessions like that”* [PT_2].

Engaging PpS in physical activity was seen by HCPs as another way of stimulating cognitive functioning post-stroke. Physical activity was described as a means by which repetitive task practice could be used to stimulate cognitive functioning: *“So again*, *even during the exercises*, *you’re working through repetitive movements*, *repetitive tasks*” [PT_1]. The progressive overload of physical activity interventions was described as having additional cognitive benefits: *“I think the research shows that*, *you know*, *that increasing your physical activity has an impact on your cognitive function*, *but also by increasing the demands of an activity*. *So by pure nature of rehabilitation… they’re having to think about how to adjust to the increased demands that you’re putting on them physically*. *So that is cognitive in itself*” [PT_2].

PpS stated that use of puzzles such as crosswords, spellings, Sudoku and computerized games could be beneficial for memory: *“Memory cards*, *puzzles*, *we do word searches and that*, *we do games with charades*. *Yeah*, *so that way*, *you kind of remember things”* [PpS_7]. HCPs, while noting the potential benefits of pen and paper type cognitive tasks, stated the need to ensure the context and meaningfulness of these tasks is communicated effectively to the PpS: *“I think you need to take the time to explain*, *the reason for [crossword puzzles or Sudoku] is to get you back driving*, *you need to be able to focus on this task and avoid whatever is dividing your attention*, *all of those kinds of things*. *Yeah*, *I don’t like doing them*, *I suppose in isolation*, *for the sake of it”* [OT_2].

### Point of readiness

#### Rehabilitation should start as early as possible

Most HCPs, particularly occupational therapists, advocated for cognitive intervention to be started as early as possible, usually defining this as when the PpS was medically stable. This could even be in the ICU setting: *“Right from the beginning*,… *these people would most likely be in ICU*. *And thankfully*, *we now have occupational therapists who are actually working in ICU… obviously [medical] stability is very*, *very important”* [OT_1]. PpS provided further perspectives into the optimal timing of cognitive intervention, having experienced how fatigue can be a barrier to engaging in cognitive intervention.

However, even when the PpS is medically stable, HCPs and caregivers should be mindful of fatigue and intervene appropriately: “*You have to be mindful if they are experiencing fatigue… and get an idea of when the best time for them to do more cognitive tasks”* [PT_5]. PpS described their difficultly to engage with HCPs due to fatigue, *“I remember a lot of occasions*, *well*, *we would be having a conversation with a few people at the table and I would end up falling asleep*. *My mind would just go completely blank*. *I wouldn’t be able to concentrate on what was going on”* [PpS_1].

Other HCPs made the point that if cognitive intervention is commenced in the acute phase post-stroke, it should be based upon psychoeducation and awareness building: *“To me [psychoeducation] is the foundation of all of the therapies really*, *it can start very early on*. *And I really think the sooner the better”* [SLT_3].

HCPs pointed out that cognitive assessment was the most essential component to begin cognitive intervention and stressed the importance of a person-centered, individualised approach to cognitive intervention: *“I think it’s very patient specific*. *I think in an ideal world*, *I think that a cognitive assessment happens*, *a detailed cognitive screen and assessment happens about six weeks post stroke*. *In an ideal world*, *I think that’s when a person is at the best place and that’s when you won’t have*, *false positive in cognitive screens*, *etc*. *And*, *and I think NICE kind of recommends that and I think that’s an ideal time to tear into it”* [OT_3].

#### Coping and adjustment

Both carers and HCPs emphasised the need for a period of adjustment to allow the PpS to adapt to life post-stroke before intervening with cognitive rehabilitation. Comparing across stakeholder groups, some PpS struggled to accept that they had a stroke, or had no insight into the occurrence of a stroke: *“He went for counseling twice… But he didn’t want it*. *When he came out of the first session*, *he said he wasn’t going back anymore*. *Because he still can’t see that he is so different from what he was”* [Carer_4]. HCPs emphasized the importance of considering the emotional aspects when rehabilitating cognitive impairment post-stroke: *“I believe that you can’t kind of separate the emotional and the coping and the adjustment side of things and*, *you know*, *behaviour from cognition*, *they’re interlinked”* [OT_3]. This period of adjustment was described as one which lacked information and support by one PpS: *“What I was very much lacking*, *and I think actually it’s even a civil rights issue*, *was information*. *Nobody told me what I could expect”* [PpS_9].

### Familiar but flexible setting is key

#### A friendly and familiar environment

The home setting was viewed favourably by most participants: *“Yeah the home is familiar to them and it’s almost an achievement to get home from hospital*. *It would be great to continue on then you know”* [Carer_2]. The home setting offers a meaningful context, demands less of a cognitive load as there is less novelty to negotiate during activities and therefore allows the PpS to engage more: *“They’re engaged in something that’s meaningful to their life*, *that therefore motivates them to continue doing it where they kind of get better feedback in their own home”* [PT_4]. HCPs stated the appropriate setting should be assessed in accordance with the profile of cognitive challenges for the PpS: *“I think if someone has more moderate or severe deficits*, *then the home environment would be better because it’s familiar because it demands less of a cognitive output*, *and so therefore*, *you know*, *a person might have capacity to actually engage more in kind of more challenging cognitive rehab”* [OT_3].

However, HCPs stated how the home setting may not always be appropriate due to inadequate levels of support in the home, *“It depends on the level of support they need*. *So it could be very challenging for the caregiver*, *especially if they’re needing 24 hour supervision*. *But certainly*, *some formal rehab setting would be definitely more useful*. *And so*, *the home could be in the medium to longer term”* [PT_4]. PpS also recognized the need for additional support and equipment in the home setting: *“I don’t have any problem with any place*, *but I’d probably sooner do it at home*. *You’re in familiar territory*. *But they wouldn’t have the equipment at home”* [PpS_6].

#### Transition across multiple settings

The majority of HCPs expressed a preference for different settings, in line with the severity of the deficits of the PpS and their rehabilitation progress, *“So I think that the initial phase*, *or kind of*, *the most intensive phase offered should be in some sort of a step down or a rehab focused facility*. *And then*, *as the person is making improvements*, *or whatever the case may be with their physical and cognitive function*, *that that would then be transitioned into their home*, *and continued from their home”* [PT_1].

The majority of participants were in agreements that rehabilitation outside of the inpatient hospital setting was best, “*So you know*, *more into the community-based*, *of course*, *is better*, *and you know*, *back to home is best”* [OT_1]. HCPs also placed an emphasis on meaningful environments to support rehabilitation needs and the importance of considering the preference of PpS *“I think that the building would have to be accessible*, *there would have to be parking*, *public transport*, *all those things*. *And I think having the capacity to also carry part of it out in their own home if they consent*, *or their work environment would be great*. *So kind of those meaningful environments”* [OT_3].

### Pragmatics of intervention delivery

#### Group vs individual

The need for individualized interventions that target improvement in specific cognitive deficits of the PpS was emphasized. Occupational therapists in particular stressed the importance of an individually tailored approach:

*“It’s very specific*, *I would say*… *because everybody is so unique*. *Yeah*, *everyone’s history*, *everyone’s personality*, *everyone’s education levels*, *everyone’s you know*, *premorbid cognition*, *everyone’s resilience*, *everyone’s mood*, *everyone’s outlook on life*. *You know*, *everyone’s family supports*, *and I suppose everyone has a different stroke as well*. *The challenge with cognitive rehab*, *and the challenge for upping our evidence-base for it and to be able to design a one size fits all*, *I think it’s an unrealistic expectation here”* [OT_3].

PpS felt that their specific needs were being met during one-to-one therapy: *“Well it’s more focused on the person*, *instead of a group*. *It can be too general [in a group] and*, *yeah I think I would prefer a one to one*, *I would feel it would be better”* [PpS_3]. However, PpS also noted the benefits of a group setting, namely motivation and peer support: “*A group is better I think*, *because you have other people to gauge your own abilities off*. *You can kind of see what everyone else is doing*. *And learn off them as well*. *Yeah*, *it’s a good motivation”* [PpS_2].

Carers provided further insight into this, recommending an individualised approach in the beginning that can be transitioned into a group format in line with the progress of the PpS:*“It would have to start out individualized… Now it could taper out then into a group and into online*. *I would think that when the person is rehabilitated cognitively*, *then online contact would be perfectly fine at that stage*, *but that’s at the end of the road*. *I think in the early stages*, *they need absolute one to one*, *to build their confidence and to give them the attention and the input and to monitor what’s happening”* [Carer_1].

#### A wide range of people can be involved

Common to all stakeholder groups was the favouring of a multidisciplinary approach to intervention delivery. PpS had confidence in a “rehabilitation team” due to their knowledge and training, *“I suppose it’s the professionals*. *So like*, *the rehabilitation team*, *I think*, *would be the best because you’d be more inclined to listen to them”* [PpS_5]. The occupational therapist was referred to as the most suitable HCP to lead this type of intervention, often as a result of their functional and pragmatic approach to intervention, *“I have to say*, *you know*, *OTs are in a great position for being multi trained*,…, *we do have such a good training in the physical side of things*, *and we also have the psychological side of things*” [OT_1]. OTs can offer a holistic approach to intervention and facilitate the transference of formal rehabilitation outcomes to the everyday life of the PpS, *“[Occupational therapists] have to have a handle on all of those exact functional attentional and memory tasks and they have a very skillful base in those issues*, *and then they have that lovely pragmatic*, *you know*, *functional approach to people”* [PT_4]. Caregivers were also in favour of an OT-led intervention, *“The occupational therapist] read him like a book*. *She was really*, *really good*, *and she educated me on a lot of things as well regarding what to do and what to be aware of*.*”* [Carer 4]

There were mixed views on the involvement of caregivers in the delivery of cognitive intervention post-stroke. The need to be cognisant of caregiver burden during a difficult time of change was identified by both caregivers and HCPs, *“I suppose the carer*, *they’re at an awful disadvantage because you’re just landed with this person*, *a different person*, *you know”* [Carer_5]. While caregiver burden must be considered, common to all stakeholders was the favouring of including caregivers in goal-setting and identifying interests of the PpS: *“Certainly*, *[carers] would be very well placed to give you some input into what the person’s interests would have been pre injury”* [PT_5]. PpS reported feeling comfortable with a caregiver as they would know their daily routine and offer a sense of familiarity: “*Well you see*, *S is my full time caregiver*, *I think the caregiver would be ideal because they’re at home with you more and they know what your day to day is”* [PpS_3].

#### Intervention length and frequency

There were varied opinions with regard to the optimal duration and frequency of cognitive intervention. Proponents of daily intervention felt that short, daily sessions would be more effective than having one long session in a week as this would make the intervention habitual and would facilitate achievement of the intended intensity: *“The reality is that cognitive rehab is all day every day*, *because you’re hoping that what you’re doing in a therapy session is carrying over into their everyday life and stimulating them*. *So you need intensity and you need it regularly”* [PT_6].

There were further varied perspectives on the duration of sessions. The shortest session length was 30 minutes, with some recommendations of sessions lasting one hour and 30 minutes long. Considerations should be given to fatigue and subsequent levels of participant engagement, while also allowing time for sufficient rest: *“If you got a 30 minute session in*, *I think you’re doing really well*. *And if you could go up to an hour I think that would be brilliant*, *but not always possible depending on somebody’s attention and fatigue”* [OT_5]. HCPs described intervention duration and frequency as being dependent on the goal of the session: *“Again it depends on what the task is*, *it depends what my session goal is*, *as well… the content of the session would dictate it”* [OT_3]. With regard to the overall period of intervention, views ranged from 8 weeks up to 12 weeks, with others adding that the duration should be as long as the person needs. One PpS referred to the permanence of having a stroke and that rehabilitation should address these lifelong needs *“you’ll never be someone who didn’t have a stroke*” [PpS_9]. This linked back to a person-centred approach and goal-setting: *“For as long as the person needs to reach the attainment of goals*, *and what they want to do and what they’re happy with”* [PT_6].

PpS, carers and HCPs emphasised the lack of review of these goals in the long-term. All participants referred to the lack of follow-up in stroke rehabilitation services in general: *“And [follow-up] is completely absent*, *you know*, *so when they leave here*, *there’s nothing*, *absolutely nothing*, *no follow up*. *There’s nothing you know”* [OT_4]. When PpS had been discharged from formal rehabilitation services, there was no review nor follow up on their progress: *“It’s 5 years now and we didn’t hear back from anybody*. *You’re put on all these lists but you get nowhere”* [Carer_5]. PpS felt isolated and unsupported post discharge due to the lack of follow-up: *“When I came out of hospital*, *there was nothing*. *After I was in the national rehabilitation hospital*, *there was nothing for weeks”* [PpS_3].

## Discussion

The current study elicited the perspectives of PpS, carers, HCPs and academics on the design and development of an intervention to rehabilitate cognitive deficits in PpS. Our findings reveal four key components of cognitive intervention design, namely (i) engagement must be meaningful, (ii) point of readiness, (iii) the importance of familiar, yet flexible settings for intervention and (iv) pragmatics of intervention delivery. Results further emphasise the importance of ensuring the meaningfulness of activities included in the rehabilitation of cognitive deficits post-stroke. While findings support the commencement of cognitive rehabilitation as soon as possible post-stroke, the capacity of the PpS to engage while allowing sufficient time to facilitate emotional adjustment was highlighted. The desired setting of cognitive intervention, while varied, emphasised the importance of a meaningful and familiar environment. Findings mirror the inconsistencies in the literature regarding the optimal timing and frequency of cognitive intervention post-stroke [35]. Factors such as physical and cognitive fatigue, as well as recommendations for an individualised approach to intervention require consideration when designing an intervention to rehabilitate cognitive deficits in PpS.

Our findings highlight that multicomponent interventions are seen as beneficial for rehabilitating cognitive deficits in PpS. A previous systematic review examined the efficacy of combined cognitive and exercise training in older adults with or without cognitive impairment [56]. Within this review, it was found that a combination of cognitive training and exercise training can be effective for improving cognitive function and functional status in older adults with and without cognitive impairment [56]. A recent RCT evaluated the effects of a combined physical exercise and cognitive training intervention in comparison to single component physical activity or cognitive interventions on outcomes of cognitive function in PpS with vascular cognitive impairment [57]. The combined intervention of physical exercise and cognitive training produced more favourable results on cognitive functions of memory and executive function in comparison to either of the single component interventions [57]. Furthermore, these effects were sustained over a long-term interval of six months follow-up [57]. Similar to these quantitative findings, the current findings support the use of multicomponent interventions in the rehabilitation of cognitive deficits post-stroke. The most consistent types of multiple component interventions identified in our findings were standardised types of cognitive rehabilitation e.g. computerised training or pen and paper tasks, in conjunction with a type of physical activity delivered as part of conventional occupational therapy or physiotherapy. As synthesised in a recent umbrella review of systematic reviews, RCTs of physical activity and cognitive rehabilitation interventions were the most commonly tested interventions to improve cognitive impairment in PpS [58]. Physical activity interventions were shown to be easily implemented in stroke services and provide an accessible and low-cost treatment that may preserve or retore cognitive function post-stroke [58]. Cognitive rehabilitation interventions were also seen to be effective on outcomes of cognitive function, and previous longitudinal evidence suggesting that such interventions may increase return to work post-stroke [59]. However, due to lack of strong evidence of combined physical activity and cognitive rehabilitation interventions, recommendations for practice could not be made [58].

Similar to inconsistencies in the literature [35], our findings highlight mixed perspectives regarding the optimal timing of cognitive intervention post-stroke. There was a strong preference to begin as soon as possible i.e., once the PpS is medically stable. However, considerations must be given to the capacity of the PpS to engage in cognitive intervention. Our findings suggest that the point of readiness of the PpS depends on multiple factors including emotional adjustment, insight into the occurrence of the stroke, as well as levels of cognitive fatigue. This perhaps suggests that a person-centred, individualised approach is more optimal. A ‘holistic wellness approach’ to stroke rehabilitation should focus on the person’s readiness to change and be individualized to their personal wants and needs [60]. This type of approach recognises the multidimensional levels of stroke rehabilitation and promotes the empowerment of the PpS, their support system and their environment [60]. In the current study, when referring to multicomponent interventions, some participants referred to these as ‘holistic’ interventions wherein the previous interests, hobbies or lifestyle of the PpS were considered and incorporated into their rehabilitation. A recent RCT showed that a multidomain lifestyle intervention was effective in the prevention or delay of cognitive impairment in elderly people at risk of dementia [61].

Our findings emphasise the home setting, as well as accessible community-based rehabilitation settings as the most favourable places to deliver cognitive intervention post-stroke. A recent qualitative interview study explored the end-user acceptance of a home-based stroke rehabilitation programme and highlighted the importance of designing programmes for holistic rehabilitation post-stroke [62]. For example, PpS who received telerehabilitation in the home to progress their physical rehabilitation reported improvements in their cognitive function and their social and emotional well-being [62]. Siemonsma et al [63] systematically reviewed the determinants of implementing home-based stroke rehabilitation. Similar to previous research [63] and mirrored in the current findings, the home setting was found to facilitate a more client-centred approach, encourage the active involvement of the PpS in rehabilitation and enhance problem-solving. In the current study, the home setting was seen to offer a real-world context wherein the individual was encouraged to practice meaningful real-world tasks. As well as meaningful engagement, the importance of collaborative goal-setting was identified as beneficial. The incorporation of specific, measurable goals in a real-world context could improve motivation and problem-solving capacities in the PpS. A recent RCT found that the use of goal-setting in people with chronic stroke improves executive function, attention, working memory and learning [64]. Goal-setting can have a favourable impact on the therapist-client relationship in stroke rehabilitation [65]. A recent systematic review of reviews highlighted the importance of the meaningfulness of goals as well as the active involvement of the client in goal-setting [66]. Often, meaningful goals were linked with the home setting wherein the PpS could actively engage with their real-life activities.

### Strengths and limitations

These findings will enable realistic plans for intervention design, considering feasibility and acceptability of any intervention, based on views from key stakeholders to rehabilitate cognitive deficits post-stroke. The use of the TIDIER checklist in the interview schedule provides a robust framework to develop a future feasibility study, specifically the ‘who, what, where, when, why and how” of a future cognitive rehabilitation intervention [51]. Furthermore, the novelty of including a breadth of rehabilitation interventions, as opposed to single-domain cognitive rehabilitation interventions in our interview schedule, will inform the development of a multicomponent intervention to rehabilitate cognitive deficits post-stroke. This adds to the literature by taking a multi-domain approach to cognitive rehabilitation as opposed to focusing on the rehabilitation of single cognitive domains as has previously been done.

This qualitative enquiry elicited rich data from wide ranging perspectives across stakeholder groups of PpS, carers, HCPs and academics across multiple disciplines involved in the provision of care for PpS with cognitive impairment. Taken together with existing quantitative evidence, these findings will inform the development of a feasibility trial, examining patient and process outcomes, to rehabilitate PpS with cognitive impairment. This study adhered to the COREQ reporting guidelines in order to ensure transparent and complete reporting of research findings. Methodological rigor was addressed by consideration of 20 critical questions to guide quality assessment of reflexive thematic analysis research [53].

The study findings should be interpreted in the context of limitations. Given that evidence from our systematic review demonstrates that interventionists were primarily HCPs including occupational therapists, speech and language therapists and physiotherapists [26], these HCPs were included in our current study. We acknowledge that the inclusion of the perspectives of physicians may have provided further insights. Given that the caregivers recruited were spouses of PpS, there is also a lack of perspectives from other informal and formal/paid caregivers which may limit the diversity of the sample. Given the remote nature of data collection, participants had to be able to communicate verbally over phone or via telecommunication platforms. This meant that some PpS with communication impairment or more severe cognitive impairment were not enabled to participate. A future feasibility study will use augmentative and alternative communication resources to be more inclusive of participants with communication deficits in the intervention. Finally, participants included those from the Republic of Ireland only and as such, findings are most applicable to the contexts of the people who took part in the study and thus, may not reflect experiences in other countries, nor be reflective of different healthcare systems that may be not as well-resourced financially.

## Conclusion

This study explored the perspectives of key stakeholders in stroke rehabilitation regarding the design and development of an intervention to rehabilitate cognitive deficits in PpS. Findings recognised the multidimensional nature of rehabilitating cognitive impairment post-stroke and highlighted the importance of facilitating meaningful engagement in intervention. The delivery of such an intervention by a multidisciplinary team, while being led by an occupational therapist was emphasised. Consistent with previous research, there was a lack of consensus regarding the optimal timing and frequency of such an intervention, which further highlights the need to include the perspectives of key stakeholders in the design and development of such an intervention. The findings of this qualitative study will be used to design a complex multicomponent intervention to rehabilitate cognitive deficits in PpS.

## Supporting information

S1 Appendix Interview guides: People post-stroke, caregivers and healthcare professionals. (DOCX)

S1 Checklist COREQ (COnsolidated criteria for REporting Qualitative research) checklist. (PDF)

## Abbreviations

COREQ: Consolidated criteria for reporting qualitative research
HCPs: Healthcare Professionals
MRC: Medical Research Council
OT: Occupational Therapist
PPI: Patient and Public involvement
PpS: Person post-stroke
PT: Physiotherapist
RCT: Randomized Controlled Trial
SLT: Speech and Language Therapist
TA: Thematic Analysis
TIDIER: Template for Intervention Description and Replication
UK: United Kingdom
