# Supplementary material for: Exploring the perspectives of key stakeholders on the design and delivery of a cognitive rehabilitation intervention for people post-stroke
Source: PLoS One. 2022 Jun 16;17(6):e0269961. doi: 10.1371/journal.pone.0269961 (PMC9202836; doi:10.1371/journal.pone.0269961)
Supplement: S1 Appendix — (DOCX) [file pone.0269961.s001.docx]

**Appendix I: Interview guides: People post-stroke, caregivers and healthcare professionals**

| **People post-stroke** | | |
| --- | --- | --- |
| **Topic** | **Questions/topics** (*prompts)* | **Rationale** |
| Introduction to study  Introduce the term “cognition” | This interview is focused on how to improve thinking and memory problems after a stroke.  Sometimes, thinking may be referred to as cognition or cognitive ability. It is how thoughts connect in your head.  For example, how you remember your phone number or where you put your car keys requires us to use our thought processes.  This includes remembering things, judging things and problem solving.  We hope to gain a better understanding of thinking abilities in people who have had a stroke.  With your insight into thinking ability after stroke, we hope to understand how we could improve this and perhaps develop a treatment that could improve thinking abilities after stroke. | - Explanation of the term cognition. - Everyday practical examples of how we use our cognitive abilities. - Explanation of the aim of this study |
| Cognitive deficits post-stroke in everyday life  Cognitive deficits in specific aspects of life, may include occupational role, previous hobbies or interests | In what way, if any, has your thinking ability changed since having your stroke?  *Are there certain things which are more difficult now?*  *Why?* | - Introducing the topic of interest - Allowing the individual post-stroke to describe changes to their thinking in their own terms and then using these terms in subsequent questions. - Some anticipated terms for cognition may include: feeling confused, reduced attention span, slowed down thinking, missing things in conversation - Using open-ended, neutral and clear questions avoiding leading questions and using familiar language (DeJonckheere and Vaughn 2019). |
| Cognitive deficits from acute care to inpatient/ outpatient rehabilitation to discharge home | In what ways has your thinking ability changed since leaving hospital and returning home?  *I’m now going to ask you more about X… (specific stage of rehabilitation) Do you remember the things you struggled most with during this point?*  *Further prompts: For example, remembering where you left certain things, focusing on conversations with family, engaging with doctors or other healthcare workers..* | Encouraging the stroke survivor to think chronologically about their rehabilitation journey. These time points may give structure to the telling of their experience. |
|  |  |  |
| ***Intervention design (Hoffman et al.’s guidelines for intervention description and replication checklist for reporting of interventions [TIDieR]) are being used to frame the next part of our discussion on the design and delivery of a future intervention to rehabilitate cognitive deficits post-stroke*** | | |
| **What (types of interventions)?** | Could you tell me about any strategies or techniques you would use/ have used in the past to help you with memory or thinking problems?  *Examples of this might include a diary, reminders in your phone, handouts, worksheets, a noticeboard, an app etc.*  *Have these been effective?*  *What kind of programme was it?*  *Who delivered it and when etc.*  *What kinds of materials were used in the programme?*  *Was goal-setting used? Were you involved in making these goals?*  If you could change one thing about the cognitive rehabilitation you have experienced, what would it be? | “What” component of TIDieR checklist  Scoping the stroke survivor’s insight into possible rehabilitation approaches for cognitive deficits  Gathering perspectives relating to intervention content, format, duration, dose, and delivery in accordance with MRC framework (Craig *et al.* 2008) |
|  | Apart from what you mentioned, what types of programmes or approaches do you think might improve memory problems after stroke?  *Have you considered that programmes such as:*   1. Being active or taking exercise   Some people stay active by:   - Gardening - Cleaning their house - Parking further away - Taking the stairs - Playing with their children  1. Doing “brain training” tasks that help your memory or overall thinking ability. These might be computer programmes, or maybe pen and paper puzzles? 2. Some types of therapy combine different forms of rehabilitation together, for example, doing some form of exercise with some form of brain training in the same session.   Other types of “combination programmes” might include:   - Programmes that address your lifestyle by helping you be more active, improving your motivation and practicing memory skills - Music therapy - Education about managing after stroke - Art therapy  1. Programmes that send electrical impulses through the brain which can increase brain activity. These types of programmes are called non-invasive brain stimulation protocols. 2. Programmes that improve everyday tasks such as dressing, cooking, and enable you to participate in social events such as hobbies or leisure activities. These types of programmes may also help you return to work or help you perform better at work. | “What” component of TIDieR checklist  Core question. Linking evidence with current practice and acceptability of proven/existing strategies.  Establishing a link between the interventions identified in our quantitative SR and our qualitative line of enquiry.  Physical activity interventions  Cognitive rehabilitation interventions  Multiple component interventions  NIBS protocols  Occupational-based interventions |
| **Who (delivery of interventions)?** | Who do you think would be most suitable to deliver a programme that helps improve memory or thinking ability after stroke?  *For example, a type of healthcare professional such as a psychologist, an OT, maybe a medical doctor?*  *Or maybe a spouse or a caregiver?* | “Who” component of TIDieR checklist  Core question. |
|  |  |  |
| **Where (setting of intervention)?** | Where do you think is the best place to provide someone after stroke with such a programme?  *Some ideas could include- classes in a community setting, individual one-to-one meetings with a healthcare professional in the home or healthcare setting, using technology to deliver this intervention to someone’s phone/tablet/computer.* | Core question. “Where” component of TIDieR checklist |
|  |  |  |
| **When?** | When is best to intervene with a person after stroke with a programme to improve thinking ability after stroke  *For example, do you think while the person is still in hospital, when they are undergoing rehabilitation, or when they are discharged home?*  *What time during the day is best?*  *How often? How many times per week? How long should a session be? Over what period of time?*  Would cognitive fatigue be an issue? | Core question. “When and how much” component of TIDieR checklist  Cognitive fatigue post-stroke is known to decrease participation in physical activities and rehabilitation (Hinkle et al. 2017).  It is important to be cognisant of the moderating effect of a stroke survivor’s capacity to engage with the intervention, considering potential fluctuating levels of fatigue |
|  |  |  |
| **Why?** | If we take the programme you mentioned earlier (say programme here), why do you think such an approach would work? | “Why” component of TIDieR checklist  Exploring the rationale, theory, or goal of the elements essential to the intervention |
|  |  |  |
| **How?** | If we take the programme you mentioned earlier (re-iterate programme here), how best do you think this would be delivered?  *Would a face-to face approach be best? Or perhaps an online approach?*  *Would it be better one-to one or in a group setting?*  *Would it be in a healthcare setting such as a hospital or in the home?* | Core question. “How” component of TIDieR checklist |
| **Tailoring and modifications** | If we take the programme you mentioned earlier (re-iterate programme here), do you think the programme was designed specifically for your needs? i.e., did it target the exact types of memory problems that you had?  *To explain further, did it change over time if you were not responding to the programme/ you did not like the programme?*  If no, (in answer to initial modification question), how do you think such a programme might tailored to your needs?  *Would strategies such as goal setting or group discussions help?* | **Tailoring component of Tidier Checklist**  Previous research has recommended that cognitive rehabilitation for individuals post-stroke be modified in accordance with the individual’s level of cognitive impairment (Cicerone *et al.* 2005).  Some types of rehabilitation are more effective for those with mild cognitive impairment compared to those with severe cognitive impairment (Cicerone *et al.* 2005). |
|  |  |  |

| **Caregivers of people post-stroke** | | |
| --- | --- | --- |
| **Topic** | **Questions/topics** (*prompts)* | **Rationale** |
| Introduction to study  Introduce the term “cognition” | This interview is focused on how to improve thinking and memory problems after a stroke.  Sometimes, thinking may be referred to as cognition or cognitive ability.  For example, how you remember your phone number or where you put your car keys requires us to use our thought processes.  This includes remembering things, judging things and problem solving.  We hope to gain a better understanding of thinking abilities in people who have had a stroke.  With your insight into the thinking ability of a stroke survivor whom you provide care for, we hope to understand how we could improve this and develop a treatment that could improve thinking abilities after stroke. | - Explanation of the term cognition. - Everyday practical examples of how we use our cognitive abilities. - Explanation of the aim of this study |
| Cognitive deficits post-stroke in everyday life  Cognitive deficits in specific aspects of life, may include occupational role, previous hobbies or interests | In what ways, if any, do you notice the thinking ability of the person you care for to have changed since their stroke?  *Are there certain things which are more difficult now?*  *Why?*  Is there anything they had done previously that they no longer do or have not returned to doing yet? | - Introducing the topic of interest - Allowing the carer to describe changes to the stroke survivor’s thinking in their own terms and then using these terms in subsequent questions. - Some anticipated terms for cognition may include: feeling confused, reduced attention span, slowed down thinking, missing things in conversation - Using open-ended, neutral and clear questions avoiding leading questions and using familiar language (DeJonckheere and Vaughn 2019). |
| Important roles in life | As a carer, what would you consider as the main things the person you care for has the most difficulty with during the day as a result of their thinking or memory problems?  *Tell me more about what you just mentioned…* | Core question. Encouraging the carer to think about all aspects of the stroke survivor’s current life and prioritize the three things affected most. This prioritization may give indication of the important areas to target in a potential intervention for cognitive deficits. |
|  |  |  |
| ***Intervention design (Hoffman et al.’s guidelines for intervention description and replication checklist for reporting of interventions [TIDieR]) are being used to frame the next part of our discussion on the design and delivery of a future intervention to rehabilitate cognitive deficits post-stroke*** | | |
| **What (types of interventions)?** | Could you tell me about any strategies or techniques you would use/ have used as a carer in the past to help memory or thinking problems?  *Examples of this might include a diary, reminders in your phone, handouts, worksheets, a noticeboard, an app etc.*  *Have these been effective?*  *What kind of programme was it?*  *Who delivered it and when etc.*  *What kinds of materials were used in the programme?*  *Was goal-setting used?* | “What” component of TIDieR checklist  Scoping the carer’s insight into possible rehabilitation approaches for cognitive deficits  Gathering perspectives relating to intervention content, format, duration, dose, and delivery in accordance with MRC framework (Craig *et al.* 2008) |
|  | Apart from what you mentioned, what types of programmes or treatments do you think might improve memory problems after stroke?  *Have you considered that programmes such as:*  Being active or taking exercise  Some people stay active by:   - Gardening - Cleaning their house - Parking further away - Taking the stairs - Playing with their children   Doing “brain training” tasks that help your memory or overall thinking ability. These might be computer programmes, or maybe pen and paper puzzles?  Some types of therapy combine different forms of rehabilitation together, for example, doing some form of exercise with some form of brain training in the same session.  Other types of “combination programmes” might include:   - Programmes that address your lifestyle by helping you be more active, improving your motivation and practicing memory skills - Music therapy - Education about managing after stroke - Art therapy   Programmes that send electrical impulses through the brain which can increase brain activity. These types of programmes are called non-invasive brain stimulation protocols.  Programmes that improve everyday tasks such as dressing, cooking, and enable you to participate in social events such as hobbies or leisure activities. These types of programmes may also help you return to work or help you perform better at work. | “What” component of TIDieR checklist  Core question. Linking evidence with current practice and acceptability of proven/existing strategies.  Establishing a link between our quantitative SR and our qualitative line of enquiry.  Physical activity interventions  Cognitive rehabilitation interventions  Multiple component interventions  NIBS protocols  Occupational-based interventions |
| **Who (delivery of interventions)?** | Who do you think would be most suitable to deliver a programme that helps improve memory or thinking ability after stroke?  *For example, a type of healthcare professional such as a psychologist, an OT, maybe a medical doctor?*  *Or maybe a spouse or a caregiver?* | “Who” component of TIDieR checklist  Core question. |
|  |  |  |
| **Where (setting of intervention)?** | Where do you think is the best place to provide someone after stroke with such a programme?  *Some ideas could include- classes in a community setting, individual one-to-one meetings with a healthcare professional in the home or healthcare setting, using technology to deliver this intervention to someone’s phone/tablet/computer.* | Core question. “Where” component of TIDieR checklist |
|  |  |  |
| **When?** | When is best to intervene with a person after stroke with a programme to improve thinking ability after stroke  *For example, do you think while the person is still in hospital, when they are undergoing rehabilitation, or when they are discharged home?*  *What time during the day is best?*  *How often? How many times per week? How long should a session be? Over what period of time?*  Does the person you care for experience a mental tiredness at times during their day? | Core question. “When and how much” component of TIDieR checklist |
|  |  |  |
| **Why?** | If we take the programme you mentioned earlier (say programme here), why do you think such an approach would work? | “Why” component of TIDieR checklist  Exploring the rationale, theory, or goal of the elements essential to the intervention |
|  |  |  |
| **How?** | If we take the programme you mentioned earlier (re-iterate programme here), how best do you think this would be delivered?  *Would a face-to face approach be best? Or perhaps an online approach?*  *Would it be better one-to one or in a group setting?* | Core question. “How” component of TIDieR checklist |
| **Tailoring and modifications** | If we take the programme you mentioned earlier (re-iterate programme here), do you think the programme was designed specifically for the needs of the stroke survivor? i.e. did it target the exact types of memory or thinking problems that they had?  Did you find that the programme was flexible in that it was easily modified or adapted to their needs throughout their rehabilitation journey?  *To explain further, did it change over time if the stroke survivor was not responding to the programme/ did not like the programme?*  Also, if it did not adapt, what was that experience like for the stroke survivor?  *Do you think they found it frustrating? Or maybe it didn’t bother them/ they didn’t think about it?*  If no (in answer to initial modification question), how do you think such a programme might tailored to your needs?  *Would strategies such as goal setting or group discussions help?* | **Tailoring component of Tidier Checklist**  Previous research has recommended that cognitive rehabilitation for individuals post-stroke be modified in accordance with the individual’s level of cognitive impairment (Cicerone *et al.* 2005).  Some types of rehabilitation are more effective for those with mild cognitive impairment compared to those with severe cognitive impairment (Cicerone *et al.* 2005). |

| **HCPs/ Academics in stroke rehabilitation** | | |
| --- | --- | --- |
| **Topic** | **Questions/topics** (*prompts)* | **Rationale** |
| Introduction to study  Introduce the term “cognition” | This interview is focused on how to improve cognitive deficits post-stroke.  Sometimes, thinking may be referred to as cognition or cognitive ability.  We hope to gain a better understanding of thinking abilities in people who have had a stroke.  With your insight into the thinking ability of a stroke survivor whom you provide care for, we hope to understand how we could improve this and develop a treatment that could improve thinking abilities after stroke. This could not happen without your insights so I really appreciate your time and expertise. | - Explanation of the term cognition. - Explanation of the aim of this study |
| Cognitive deficits post-stroke in the everyday practice of a HCP  Cognitive deficits in specific aspects of life, may include occupational role, previous hobbies or interests | Could you tell me a little about your caseload and the usual patients you see?  What do you understand by the term ‘cognition?’  *Prompt- other types of cognitive abilities would include neglect, perception, executive function, processing speed etc etc*  Tell me about the main cognitive issues you observe in your stroke patients…  *Are there certain things which are more difficult now?*  *Why?*  *Is there anything they had done previously that they no longer do or have not returned to doing yet?*  For patients attending your service, what do you think influences their engagement with cognitive rehabilitation post-stroke? | - Opportunity for HCPs to warm-up and give insight into their practice - Allowing the HCP to describe changes to the stroke survivor’s thinking in their own terms and then using these terms in subsequent questions. - Some anticipated terms for cognition may include: feeling confused, reduced attention span, slowed down thinking, missing things in conversation - Using open-ended, neutral and clear questions avoiding leading questions and using familiar language (DeJonckheere and Vaughn 2019).   Encouraging the HCP to discuss factors that influence engagement with rehabilitation. |
|  | What key things help people to improve their cognitive ability post-stroke?  *Examples - provision of information and support regarding the meaning and management of cognitive deficits can help?*  *What do you think of follow-up services for cognitive deficits post-stroke?*  What do you perceive as barriers to these patients improving their cognitive ability?  Are these types of rehabilitation interventions emphasized as an important component of patient care post-stroke? Do you witness individuals understanding the type of treatment for cognitive deficits? Or maybe adherence can be an issue?  Is it sometimes difficult for stroke survivors to talk about their memory deficits? | Exploration into the potential barriers and facilitators to the rehabilitation of cognitive deficits post-stroke  Facilitators identified in the literature to date:  continual access to information and support to ensure prompt and timely diagnosis of post-stroke dementia  adequate follow-up in the community   - Stronger links between specialist and community teams could help identify those at-risk and assist in targeted cognitive assessment and follow-up (Tang *et al.* 2019).   Barriers identified in the literature to date:  1) Less focus on memory and cognition in post-stroke care; 2) Difficulties bringing up memory and cognitive problems post-stroke; 3) Lack of clarity in current services; and, 4) Assumptions made by healthcare professionals introducing gaps in care (Tang *et al.* 2019). |
| ***Intervention design (Hoffman et al.’s guidelines for intervention description and replication checklist for reporting of interventions [TIDieR]) are being used to frame the next part of our discussion on the design and delivery of a future intervention to rehabilitate cognitive deficits post-stroke*** | | |
| **What (types of interventions)?** | Could you tell me about any strategies or techniques you would use/ have used as a HCP in the past to help memory or thinking problems?  *Have these been effective?*  *What kind of programme was it?*  *Who delivered it and when etc.*  *What kinds of materials were used in the programme?* | “What” component of TIDieR checklist  Scoping the HCP’s insight into possible rehabilitation approaches for cognitive deficits.  Gathering perspectives relating to intervention content, format, duration, dose, and delivery in accordance with MRC framework (Craig *et al.* 2008) |
|  | Apart from what you mentioned, in an ideal world, what features would an intervention to rehabilitate cognitive deficits in stroke survivors include?  *Have you considered that programmes such as: definitions of programmes given in Appendix A below.*  Being active or taking exercise  Some people stay active by:   - Gardening - Cleaning their house - Parking further away - Taking the stairs - Playing with their children   Doing “brain training” tasks that help your memory or overall thinking ability. These might be computer programmes, or maybe pen and paper puzzles?  Luminosity  Happy neuron  Some types of therapy combine different forms of rehabilitation together, for example, doing some form of exercise with some form of brain training in the same session.  Other types of “combination programmes” might include:   - Programmes that address your lifestyle by helping you be more active, improving your motivation and practicing memory skills - Music therapy - Education about managing after stroke - Art therapy   Programmes that send electrical impulses through the brain which can increase brain activity. These types of programmes are called non-invasive brain stimulation protocols.  Programmes that improve everyday tasks such as dressing, cooking, and enable you to participate in social events such as hobbies or leisure activities. These types of programmes may also help you return to work or help you perform better at work. | “What” component of TIDieR checklist  Core question. Linking evidence with current practice and acceptability of proven/existing strategies. The intervention content prompt is from our unpublished SR on rehabilitation interventions which improve cognitive deficits in individuals post-stroke.  Establishing a link between our quantitative SR and our qualitative line of enquiry.  Physical activity interventions  Cognitive rehabilitation interventions  Multiple component interventions  NIBS protocols  Occupational-based interventions |
| **Who (delivery of interventions)?** | Who do you think would be most suitable to deliver a programme that helps improve cognitive deficits or thinking ability after stroke?  *For example, a type of healthcare professional such as a psychologist, an OT, maybe a medical doctor?*  *Or maybe a spouse or a caregiver?* | “Who” component of TIDieR checklist  Core question. |
|  |  |  |
| **Where (setting of intervention)?** | Where do you think is the best place to provide someone after stroke with such a programme?  *Some ideas could include- classes in a community setting, individual one-to-one meetings with a healthcare professional in the home or healthcare setting, using technology to deliver this intervention to someone’s phone/tablet/computer.* | Core question. “Where” component of TIDieR checklist |
|  |  |  |
| **When?** | When is best to intervene with a person after stroke with a programme to improve thinking ability after stroke?  *For example, do you think while the person is still in hospital, when they are undergoing rehabilitation, or when they are discharged home?*  *What time during the day is best?*  *How often? How many times per week? How long should a session be? Over what period of time?* | Core question. “When and how much” component of TIDieR checklist |
| **Cognitive fatigue** | Would cognitive fatigue be an issue?  Does the person you care for experience a mental tiredness at times during their day?  Tell me about the specific kinds of things you notice that make them most tired?  *Why do you think this is?*  *When does this usually happen/ affects them the most?*  As a HCP, have you any techniques you use to help with this tiredness and still manage activities in some form as you have described? | Cognitive fatigue post-stroke is known to decrease  participation in physical activities and rehabilitation (Hinkle et al. 2017).  It is important to be cognizant of the moderating effect of a stroke survivor’s capacity to engage with the intervention, considering potential fluctuating levels of fatigue. |
| **Why?** | Why do you think people improve once they receive the X intervention  *Why do you think they work?* | “Why” component of TIDieR checklist  Exploring the rationale, theory, or goal of the elements essential to the intervention |
|  |  |  |
| **How?** | If we take the programme you mentioned earlier (re-iterate programme here), how best do you think this would be delivered?  *Would a face-to face approach be best? Or perhaps an online approach?*  Would it be better one-to one or in a group setting?  *Would it be in a healthcare setting such as a hospital or in the home?* | Core question. “How” component of TIDieR checklist |
| **Tailoring and modifications** | If we take the programme you mentioned earlier (re-iterate programme here), do you think the programme was designed specifically for the needs of the stroke survivor? i.e. did it target the exact types of memory or thinking problems that they had?  *Did you find that the programme was flexible in that it was easily modified or adapted to their needs throughout their rehabilitation journey?*  Also, if it did not adapt, what was that experience like for the stroke survivor?  If no (in answer to initial modification question), how do you think such a programme might tailored to your needs?  If you could change one thing about the cognitive rehabilitation you provide, what would it be? | **Tailoring component of Tidier Checklist**  Previous research has recommended that cognitive rehabilitation for individuals post-stroke be modified in accordance with the individual’s level of cognitive impairment (4).  Some types of rehabilitation are more effective for those with mild cognitive impairment compared to those with severe cognitive impairment (Cicerone *et al.* 2005). |
| **Feasibility** | What would influence the uptake of an intervention to rehabilitate cognitive deficits in your service?  What would support uptake?   - *HCP confidence in delivering a programme* - *Receive training on the delivery of such an intervention* - *Appropriate equipment/ resources on site*   What would inhibit uptake?  *Potential barriers*   - *Increased workload (training clinicians/ patients)* - *Difficulty implementing a potential novel type of intervention into workflow* - *Cost* - *Data management/ confidentiality/ privacy* | Core question: Gaining insight into the feasibility/acceptability of any potential intervention clearly from this group’s (those to deliver it) perspective. |
| **Closing of interview:** | Thank you so much for your time today. That brings us to the end of our time. This discussion was very valuable and we have gained a lot of useful information.  Before we finish, is there anything else that hasn’t come up yet that you would like to talk about? | These are the main things that came up: [*brief summary*]  If something comes up after you leave, please get in contact. My details are on the information sheets or you can get in contact with [Site-specific person].  For anonymity, I won’t share a transcript of the focus group. If anyone is interested, I can send a summary of the results. Thank you again for your time and valuable insights. |

**References:**

1. DeJonckheere M, Vaughn LM. Semistructured interviewing in primary care research: a balance of relationship and rigour. Fam Med Community Health. 2019 Mar 8;7(2):e000057–e000057.

2. Hinkle JL, Becker KJ, Kim JS, Choi-Kwon S, Saban KL, McNair N, et al. Poststroke fatigue: emerging evidence and approaches to management: a scientific statement for healthcare professionals from the American Heart Association. Stroke. 2017;48(7):e159–70.

3. Craig P, Dieppe P, Macintyre S, Michie S, Nazareth I, Petticrew M. Developing and evaluating complex interventions: the new Medical Research Council guidance. Bmj. 2008;337:a1655.

4. Cicerone KD, Dahlberg C, Malec JF, Langenbahn DM, Felicetti T, Kneipp S, et al. Evidence-based cognitive rehabilitation: updated review of the literature from 1998 through 2002. Archives of physical medicine and rehabilitation. 2005;86(8):1681–92.

5. Tang EY, Price C, Stephan BC, Robinson L, Exley C. Post-stroke memory deficits and barriers to seeking help: views of patients and carers. Family practice. 2019;36(4):506–10.
