# Supplementary material for: Exploring the perspectives of key stakeholders on the design and delivery of a cognitive rehabilitation intervention for people post-stroke
Source: PLoS One. 2022 Jun 16;17(6):e0269961. doi: 10.1371/journal.pone.0269961 (PMC9202836; doi:10.1371/journal.pone.0269961)
Supplement: Attachment — Submitted filename: Response to reviewers.docx [file pone.0269961.s003.docx]

| **No. of comment** | **Reviewer 1** | **Response** |
| --- | --- | --- |
| 1 | **Dear authors Thank you for this manuscript, it is pertinent, interesting and largely well structured. I am making mostly minor rewording/restructuring suggestions with some requests for further detail on methods, for clarity, completeness and readability.** | Thank you for your comprehensive review of this manuscript. Please see point-by-point responses below. Please note that original line numbers have changed since revisions have been made to the main manuscript.  We have outlined all changes made here as clearly as possible with changes outlined in accordance with:  “Section of manuscript, Page X, Line X-X.” |
| 2 | **Abstract and 583-586 ‘Evidence based and stakeholder informed’ is implicit. Suggest simply replace with 'Taken together with existing quantitative evidence, these findings will inform the development of a feasibility trial, examining patient and process outcomes, to rehabilitate cognitive deficits poststroke.'** | Thank you for this insight. We have amended this in the abstract. Please see Abstract, Page 2, Line 22 to 24.  We have also amended this line in the discussion. Please see Discussion, Page 28, Line 19-21. |
| 3 | **63: “varying incidence rates between 20-80% of PpS” - clarify this is stroke of any aetiology?** | Thank you for requesting clarity on this. We have confirmed the aetiologies of stroke with regard to references (3-5) which are as follows:  (3) = ischaemic stroke only  (4) = first-ever cerebral stroke, either ischaemic or haemorrhagic  (5)= Either ischaemic stroke or intracerebral haemorrhage  We have added to the manuscript to reflect the mixed aetiology of the incidence rates between 20-80% of PpS.  Please see Introduction, Page 4, Line 4-5. |
| 4 | **65-66: “Data from a prospective population-based stroke register found the prevalence of cognitive deficits in 22% of people at five years post-stroke and 21% of people at 14 years post-stroke (7).”  Needs rewording e.g. the prevalence of cognitive deficits at 5 years post stroke was 22%...** | Thank you. We have reworded this sentence as suggested. Please see Introduction, Page 4, Line 6-8. |
| 5 | **67: “Cognitive impairment post-stroke is shown to be independently associated” Reword: has been shown to be independently associated OR is independently assoc...** | This has been re-worded to clarify the association between cognitive impairment and stroke.  Please see Introduction, Page 4, Line 9. |
| 6 | **75-76: “factors that could facilitate empowerment and enable PpS to regain control over their everyday life” Define empowerment, or leave it out as I suspect ‘regain control over’ covers it.** | Thank you. We agree that the word ‘empowerment’ is repetitive here and does not add to this sentence. We have taken it out and re-structured the wording of the sentence.  Please see Introduction, Page 4, Line 17. |
| 7 | **79-81: This has been superseded by a Stroke Association/James Lind Alliance PSP (2021) which puts cognitive research at #2, after psychological** | Thank you. We have amended this in accordance with the most up to date publication by the James Lind Alliance and referenced the latest publication. We have also changed the wording of this sentence to show how cognition is “among the top priorities for stroke research”. Please see Introduction, Page 4, Line 20-22. |
| 8 | **81-82: I would leave this sentence out here as you discuss/cite stakeholder involvement later** | Thank you. This sentence “Understanding the perspectives of key stakeholders is vital to inform the design and development of an intervention to rehabilitate cognitive deficits in PpS” has been taken out. |
| 9 | **98-99: so did (27) only focus on a particular intervention type? That would make the distinction from present study clearer. What did (27) conclude?** | Thank you for requesting clarity on this. Yes (27) focused on psychological interventions only. We have clarified this in the manuscript. We have also added detail on the conclusion from (27) and highlighted the novelty of the current qualitative study which focuses on a breadth of rehabilitation interventions delivered by a range of allied health professionals. Please see Introduction, Page 5, Line 20-26, and Page 6, Line 1-3. |
| 10 | **96-107 I would reorganise this para to avoid repetition. “Cog rehab is defined as…” then your systematic review of 64 studies, then say the previous quali enquiry only focused on a narrow remit (if it did), hence the need for present quali study looking at wider range of interventions etc.** | Thank you. In conjunction with additions to the manuscript in response to comment 9 above, this section has been re-worded as recommended by comment 10. Please see Introduction, Page 5, Line 12-26, and Page 6, Line 1-3. |
| 11 | **109-110 can you give an example of negative effects from the cited reference** | We have provided examples of such negative effects.  Please see Introduction, Page 6, Line 6. |
| 12 | **115 HCPs – use acronym from the first instance of healthcare professional in main text** | Thank you. This has been amended. Please see Introduction, Page 6, Line 11. |
| 13 | **131 these findings – clearer to say “the findings from the present study”** | This has been re-worded.  Please see Introduction, Page 7, Line 7-8. |
| 14 | **Sampling and recruitment A brief definition of Information power would be helpful.**  **Was the information leaflet sent at the same time as the invitation letter?**    **How long did participants have to consider before consent was sought?** | Thank you. We have now included a brief definition of information power. Please see Methods, Page 7, Lines 24-26.  We have clarified the timing regarding the sending of the recruitment letter and the participant information leaflets. Please see Methods, Page 8, Line 6-8.  We have clarified how long participants had to consider participation before consent was sought. Please see Methods, Page 8, Line 9-10. |
| 15 | **174 capacity assessed on a continual basis – surely not for a one-off interview? Capacity wouldn’t be expected to fluctuate during the course of a single interview.**  **How did you assess capacity?** | Thank you for this question. That we assessed on a continual basis refers to capacity being established initially and then reviewed throughout participation in the interview i.e. immediately after the interview had been conducted. We did not following up at a later date.  We have re-worded this section to demonstrate how capacity to consent was determined initially by the gatekeeper and subsequently checked by MOD before participation in the interview. Please see Methods, Sampling and Recruitment, Page 9, Line 17-21. |
| 16 | **Methods Although you mention reflexive thematic analysis and methodological rigour several times, I don’t find this convincing as there is no evidence of reflexivity – no fieldnote excerpts. And memo-ing is not in and of itself reflexive – depends what you’re noting down. How did your biases affect data collection and analysis/interpretation? I appreciate you may not have the space to discuss in any detail.** | Thank you for requesting further insight on this.  We have added further detail on the reflexive approach to thematic analysis to highlight the researcher’s active role in knowledge production. Please see Methods; Data Analysis, Page 11, Line 9-11 and Line 13-15. |
| 17 | **Data analysis I found this section repetitive…peer debriefing, continuous discussion, debriefing mentioned again in the six stages section – are these the same conversations? You mention fieldnotes/diary twice.** | Thank you. In conjunction with changes made to Data Analysis in comment 16, this section has been re-worded with repetition of peer debriefing, continuous discussion, fieldnotes/ diary removed. Please see Methods; Data Analysis, Page 11, Line 7-15. |
| 18 | **Findings 440: who thought interventions should be OT-led – anyone other than the OT?** | Thank you for this insight. There were multiple sources of data on this point and information about these have been added  In Results section, Page 22, Line 26-Page 23, Line 1-2, a physiotherapist thought that such an intervention should be OT-led:  We have added in a further quote to illustrate how caregivers were also in favour of an OT-led intervention. Please see Results, Page 23, Line 2-5. |
| 19 | **250-272 This section is repetitive e.g. ‘context-specific, meaningful goals were noted to promote engagement’ is there in 2 or 3 different forms.** | Thank you. We have cut out repetition of meaningful engagement. Please see Results, Page 15, Line 14-16. |
| 20 | **255 not obvious what the intervention would be, what do the HCPs do to ‘engage’ PpS?** | Upon reflection, we believe the term ‘participation’ is a more accurate description as opposed to engagement, which is more complex to define as pointed out by the reviewer. The term ‘engagement’ has been replaced with ‘participation’. Please see Results, Page 15, Line 17. |
| 21 | **288-289 Probably some/all were not realistically going to get back to their pre-stroke ‘normal’** | Thank you. We agree that this sentence was mis-leading. We have re-worded the benefits associated with occupational intervention to reflect the data more clearly.  Please see Results, Page 16, Lines 22-23. |
| 22 | **296 'benefits of an interdisciplinary approach in the delivery' - correct to 'in delivering this type of intervention'** | Thank you. This has been re-worded as suggested. We believe the text reads more clearly now.  Please see Results, Page 17, Line 3-4. |
| 23 | **312-319 Overlap with/repetition of theme from your first section – context and meaningfulness** | Thank you for highlighting this. This was also highlighted in comment 19, where we have since re-phrased the term meaningful engagement to understanding the relevance of tasks. Please see Results, Page 15, Line 14-16.  We believe that the reference to meaningful participation in this final section of the subtheme “Variety of intervention can stimulate a real-world context”, shows coherence between the theme and its underlying subthemes. Therefore, we have decided to keep this section. We are happy to revise this if the reviewer deems necessary. |
| 24 | **Discussion 535 clearer to say ‘lack of strong evidence of combined PA and cog rehab interventions’ if that’s what you mean** | Thank you for this suggestion. We agree this re-wording more clearly demonstrates our discussion point relating to combined physical activity and cognitive rehabilitation interventions. We have re-phrased this sentence.  Please see Discussion, Page 26, Line 15-16. |
| 25 | **543-545 largely repeats first sentence in para** | Thank you for pointing out this repetition. This sentence has been removed. |
|  |  |  |

| **No. of comment** | **Reviewer 2** | **Response** |
| --- | --- | --- |
| 1 | **Thank you for the opportunity to review this paper reporting an approach to developing a multidisciplinary intervention to rehabilitate cognitive deficits in people after a stroke: a relevant topic. A qualitative study was undertaken through interviews with people post-stroke, carers and rehabilitation healthcare professionals. The findings were mixed with different views expressed on topics such as when to initiate rehabilitation. The authors identified four themes for the design and development of an intervention.** | Thank you for your comprehensive review of this manuscript. Please see point-by-point responses below. Please note that original line numbers have changed since revisions have been made to the main manuscript.  We have outlined all changes made here as clearly as possible with changes outlined in accordance with:  “Section of manuscript, Page X, Line X-X.” |
| 2 | **Reference 28 is cited several times in the paper. For example, on page 5, “as evidenced from a previous systematic review (28). O’ Donoghue et al. (28) conducted a systematic review of 64 studies addressing any type of non-pharmacological rehabilitation intervention which may improve cognitive function in people post-stroke.” and “Taken together with quantitative evidence from our systematic review (28)…” Reference 28 is to the protocol for a systematic review written by the first author of this paper. The same protocol paper is cited again as reference 49, “The interview guide, (supplementary material), was informed by our previous systematic review (49)…”. Based on Google Scholar and PubMed searches, the paper resulting from the protocol does not appear to have been published,. The results reported in the current paper from reference 28 are thus not in the public domain. This has implications for both the future publication of the paper based on the protocol as some of the results will have been presented in this paper if it is accepted, and the veracity of the information provided in this paper is in question if the paper, references 28 and 49, has not been published** | Thank you for this insight. The systematic review and meta-analysis results is published in Stroke AHA.  We have now replaced the SR protocol paper reference with the published SR reference:  26. O’Donoghue M, Leahy S, Boland P, Galvin R, McManus J, Hayes S. Rehabilitation of Cognitive Deficits Poststroke: Systematic Review and Meta-Analysis of Randomized Controlled Trials. Stroke. 2022;STROKEAHA-121.  Please see Introduction, Page 5, Line 12. |
| 3 | **The sample size of 30 interviewees was guided by ‘information power’ as defined by Malterud, who in explaining the process states, “For each of these items, we have proposed dimensions along a continuum where researchers are invited to position themselves and their study to assess an approximate number of participants needed for responsible analysis. We argue that such an assessment should be stepwise revisited along the research process and not definitely decided in advance. In this way, recruitment can be brought to an end when the sample holds sufficient information power. Still, the model may offer support also in the initial planning of a qualitative interview study.” There is no mention of stepwise analysis of interview findings in the methods section. Was this done? If not, how was the sample size of 30 determined.** | Thank you for this insight regarding “information power". We followed a stepwise analysis in accordance with Malterud *et al.* (2016) model of information power. We have added detail to the manuscript to expand on this. Please see Results, Page 12, Line 16-26. |
| 4 | **One of the inclusion criteria for people post-stroke was “Able to verbally communicate over the phone/ via telecommunication platform” (Line 155). In lines 179-180, it is stated that “In cases where the individual post-stroke was unable to effectively communicate in a semi structured interview setting, we informed participants that proxy respondents may be used.” If they were not able to communicate effectively, why were they included and interviewed?** | Thank you for this comment.  We had initially included detail around the use of proxy respondents to be as inclusive as possible in facilitating people with communication difficulties post-stroke to participate in this qualitative interview study. However, given that the people post-stroke recruited to this study were able to verbally communicate over the phone/ via telecommunication platform, we did not end up using proxy respondents in this study.  As highlighted by the reviewer, we can see that the text regarding the use of proxy respondents is contradictory to our inclusion criteria.  We have removed detail on proxy respondents in the manuscript as they were not used in the study. |
| 5 | **The five carers interviewed were the wives of five of the people post-stroke who were interviewed. What is the effect of this on the spectrum of information that can be gathered from these five pairs? Does it lessen the ‘information power’?** | We acknowledge the valuable insights that spouses, as caregivers of people post-stroke, can provide. We also acknowledge this as a limitation in terms of the diversity of the sample and that it may lessen information power. Detail on this has been added. Please see Strengths and Limitations, Page 29, Line 5-7. |
| 6 | **Granted, they will know how the stroke event has changed their partner, but what effort was made to find carers who were not partners of the patient?** | As described in Methods, Page 8, Line 23-25, we reached out to the Carers Association Limerick to maximise diversity in the carers group. Given their busy workload and during Covid-19 restrictions, unfortunately we did not manage to recruit caregivers via this recruitment path. Therefore, the caregivers we recruited were spouses of people post-stroke. We have added in further detail on two caregivers from the Carers Association Limerick, declined to interview. Please see Results, Page 13, Line 3. |
| 7 | **What additional insight may they have provided?** | We acknowledge that carers who were not partners of the person post-stroke could provide additional insights. We have noted this as a limitation. Please see Strengths and Limitations, Page 29, Line 5-7. |
| 8 | **Methods, line 178: to what does the abbreviation PPI refer?** | PPI refers to Patient and Public Involvement. We have clarified this in the manuscript. Please see Methods, Page 10, Line 1. |
| 9 | **Limitations: the absence of physicians in the study is noted, but the reason for their exclusion, the desire “explore the management of cognitive impairment post-stroke using rehabilitation interventions with no emphasis on pharmacological interventions” appears ill-founded unless doctors are to be specifically excluded from the multidisciplinary rehabilitation process. Doctors may well prescribe medications irrespective of the rehabilitation plan, especially if they are not part of the process.** | Yes, while the absence of physicians is acknowledged as a limitation, we agree that our reasoning for this may be ill-founded. We have removed text in the manuscript detailing that physicians were not included as we aimed to “explore the management of cognitive impairment post-stroke using rehabilitation interventions with no emphasis on pharmacological interventions only”.  However, physicians do not specifically deliver rehabilitation interventions and so, a more clinically informed rationale for their exclusion goes back to our research aim which is “to elicit perspectives on the delivery of cognitive intervention post-stroke.” Detail on the role of AHPS in the delivery of rehabilitation interventions post-stroke has been added to the manuscript. Please see Strengths and Limitations, Page 29, Line 1- 4. |
| 10 | **Limitations: it is noted the findings may be specific to the Republic of Ireland and may not be representative of all patients-post stroke. It should also be noted that any programme which is developed from this study will also be relevant to similar health systems with similar resources, both human and financial, but not necessarily applicable to less-resourced settings.** | We have noted this as a limitation also. Please see Discussion; Strengths and Limitations, Page 29, Line 12-13. |
| 11 | **In Figure 1, what status was afforded to, ‘individual interests of persons must be encouraged’ and ‘variety of interventions can stimulate a real-world context’ are they subthemes or individual themes that were not chosen to be included among the four key themes and if so, why?** | These are subthemes which fall under the main theme “Engagement must be meaningful”. We have added a sentence to detail the layout of main themes with their corresponding subthemes. Please see Results, Page 15, Line 3-4. |
| 12 | **Data collection, lines 198-204: is the information provided about the lead author relevant to the paper?** | Thank you. In line with Item 8 of the COREQ checklist:  **Item 8:** **Interviewer characteristics**: What characteristics were reported about the inter viewer/facilitator? e.g. Bias, assumptions, reasons and interests in the research topic?   - We believe that the information regarding the lead author is relevant. We are willing to take this information out if deemed unnecessary. |
| 13 | **The authors have chosen to identify those who were involved in the various aspects of the study. The lead author is identified several times. PB, SH and DL are identified with respect to coding, line 216, to peer debriefing sessions with the lead author in line 230 and PB for other actions in lines 217 and 225. What roles did the other four authors play in the study?** | Thank you for this comment. The role of all authors in the manuscript preparation and revision is articulated in a paragraph following the conclusion.  Please see Author Contributions, Page 30, Line 5-9. |
|  |  |  |

| **No. of comment** | **Reviewer 3** | **Response** |
| --- | --- | --- |
| 1 | **Reviewer #3: The manuscript is a report of a qualitative analysis of 30 interviews with stakeholders involved in delivery or receipt of cognitive rehabilitation post-stroke. It is stated that a goal of the manuscript is to inform the development of an evidence based and stakeholder informed feasibility trial. However because little detail is provided about how the results are applied to that goal the manuscript does not seem to have helped achieve that milestone.** | Thank you for your comprehensive review of this manuscript. Please see point-by-point responses below. Please note that original line numbers have changed since revisions have been made to the main manuscript.  We have outlined all changes made here as clearly as possible with changes outlined in accordance with:  “Section of manuscript, Page X, Line X-X.”  We had initially included how findings will enable realistic plans for intervention design, considering feasibility and acceptability of the intervention, based on views from key stakeholders to rehabilitate cognitive deficits post-stroke.  We have added further detail on how the results of the manuscript are applied to inform the feasibility study as follows:  We have added detail on the use of the TIDIER framework (Template for intervention description and replication) which guided our interview schedule. The TIDIER framework relates directly to the information needed to inform the intervention to be delivered in the feasibility trial, specifically the “who, what, when, why, why and how” of intervention design. Please see Methods, Page 10, Line 13-16 and Strengths and Limitations, page 28, Line 8-10.  We have also added detail on the novelty of this research which adopts a multi-domain approach to cognitive rehabilitation as opposed to focusing on domain-specific cognitive interventions as has previously been done. Furthermore, we have focused on a breath of rehabilitation interventions, delivered by a range of healthcare professionals, not only psychological interventions. Please see Introduction, Page 5, Line 14-19. |
| 2 | **The findings are of interest, yet it is not clear whether the findings are new or how they add to the literature, so a perspective on how the findings would influence the development of an intervention or trial would be a useful addition.** | Thank you for this comment. As outlined in our response to Reviewer 2, comment 1, we have added detail on the TIDIER framework to guide our interview schedule, specifically the ‘who, what, where, when and how” of intervention design.  We have also added further detail on the multi-domain approach to cognitive rehabilitation and the breadth of rehabilitation interventions which will inform a future feasibility trial. Please see Introduction, Page 5, Line 14-19. Please see Methods, Page 10, Line 13-16 and Strengths and Limitations, Page 28, Line 8-10.  We have also outlined novelty of the current study in contrast to previous literature. For example, we have added detail on the conclusion from (28) and highlighted the novelty of the current qualitative study which focuses on a breadth of rehabilitation interventions delivered by a range of allied health professionals. Please see Introduction, Page 5, Line 26- Page 6, Line 1-3. |
| 3 | **The figure illustration has arrows in the wrong place for the subtheme for theme #1.** | Thank you for pointing this out. This has been amended.  Please see Figure 1, supplementary material. |
| 4 | **The Methods describe that sample size is achieved with Information Power. I would suspect that this was achieved, however the authors do not provide any description for why the study met the criteria underlying the concept. A careful defense of the broad nature of the different types of interventions that would be under consideration here and the disparate people interviewed because those would tend to diminish an argument that Information Power was achieved with the moderate number of interviews.** | Thank you for raising this point. Please see our response to Reviewer 2, Comment 3, who raised a similar query.  Moreover, in relation to the study sample size and information power, the higher number of of HCPs interviewed (n=15) gave in-depth responses regarding the design and delivery of a future intervention from their clinical expertise. On the other hand, the contextual factors of the intervention were derived mainly from PpS and carers e.g. point of readiness, emotional adjustment post-stroke, caregiver burden. Please see Results, Page 12, Line 17-26. |
| 5 | **The recruitment is not clearly described? Also, the number invited / declined to the interview was not found in the manuscript.** | Thank you for requesting clarity on this. We have added further detail on our recruitment strategy. Please see Methods; Sampling and Recruitment, Page 8, 5-10.  We have also added detail on the number who were invited and who declined to interview as follows:  People post-stroke who declined to interview n=1  Caregivers who declined to interview n n=2  HCPs who declined to interview n=0  Please see Results, Page 12, Line 1-2. |
| 6 | **There is no description of the length of interviews (in a unit of time), the length of the transcripts (in pages, quotes, or words). It is thus difficult to judge whether the sample was sufficient.** | Thank you for this comment. We have added the interview length of each participant in a separate column labelled “interview length” in table 1, table 2 and table 3 respectively. We hope this, along with added detail on our stepwise analysis of information power, gives clarity on our sample size.  Please see Results, Page 13-14, Table 1, Table 2, Table 3. |
| 7 | **The manuscript should include a box or table with a listing of acronyms used in the text and their meanings.** | Thank you. A listing of acronyms has been added. Please see Page 3, Line 1-13. |
| 8 | **P17 – the author state that “majority of participants were in agreement that community-based best” but the following quote used as an example states “back to home is best” – The discrepancy needs to be resolved or explained.** | Thank you for this comment. This section has been edited to reflect the content of the quotations provided. Please see Results, Page 21, Line 4-5. |
| 9 | **­­­­There are some statements that are confusing e.g., P20 “with session length varying from 30 minutes up to one hour and 30 minute long sessions.”** | Thank you for requesting clarity on this. Detail regarding session length has been clarified. Please see Results, Page 24, Line 2-3. |
| 10 | **There are several supplemental files provided with the manuscript, but it seemed that only one was referenced in the manuscript. (i.e. p7: “Interview scripts are available as supplemental material”).** | Thank you for this comment. Participant demographic tables (Table 1, table 2 and table 3) were initially included as supplemental files. We have since included these tables in the main manuscript in response to comment 11 below. Please see Results, Page 13 and 14, Table 1, table 2, table 3.  Therefore, the only supplemental files now are the interview scripts, the COREQ checklist and Figure 1.  We hope this clarifies your query in relation to supplemental files. |
| 11 | **The participant information provided in a supplementary file, may be more accessible and informative if summarized in the result section.** | Thank you for this comment. We have summarized the participant demographics in the following tables: Table 1 (demographics of people post-stroke), Table 2 (demographics of caregivers) and Table 3 (demographics of HCPs) in the results section. Please see Results, Page 13 and 14, Table 1, table 2, table 3. |
| 12 | **It is not clear what the authors intent is for the purpose of the supporting file ‘Implications for Rehabilitation’. This information should be referenced in the text or incorporated in the manuscript.** | Thank you for requesting clarity on this. We have integrated the implications for rehabilitation into our discussion, and this is no longer a supporting file. Please see Conclusion, Page 29, Line 19-26 and Page 30, Line 1-2. |

| **No. of comment** | **Reviewer 4** | **Response** |
| --- | --- | --- |
| 1 | **Reviewer #4: Thank you for the opportunity to review this paper on the perspectives of cognitive rehabilitation after stroke. I make the following comments and queries for your consideration:** | Thank you for your comprehensive review of this manuscript. Please see point-by-point responses below. Please note that original line numbers have changed since revisions have been made to the main manuscript.  We have outlined all changes made here as clearly as possible with changes outlined in accordance with:  “Section of manuscript, Page X, Line X-X.” |
| 2 | **Abstract 1. the translation of the findings into an eventual intervention is welcomed.** | Thank you for this comment. |
| 3 | **2. perhaps change the term to cognitive impairment rather than deficit? Introduction** | Thank you for raising this point. We have changed the term to cognitive impairment. Please see Introduction, Page 4, Line 4. |
| 4 | **3. excellent scene setting and justification up to page 5; then the text goes specifically into memory having just justified a more global perspective in this study?** | Thank you. We have added more detail on the burden of general cognitive impairment post-stroke in relation to reduced quality of life and the cognitive impairment being among the highest proportion of unmet needs this population. Please see Introduction, Page 6, Line 13-19. |
| 5 | **4. and the final paragraph in the intro also is a bit disjointed. Is the point that you want to produce an evidence-based intervention? then you can justify by pointing out that an evidence base includes - effectiveness evidence, expert opinion and patient values and preferences. so there is your justification. That may be what the MRC says but i am not familiar with whereas everyone knows Sackets triangle.** | Thank you for this insight. We have re-structured the final paragraph in the introduction to communicate a clearer message for evidence-based intervention development. Please see Introduction, Page 6, Line 23-26, and Page 7 Line 1-11.  We agree the elements of Sacket’s triangle are key to inform the development of an evidence-based intervention. The MRC framework centres on these components, specifically in the development phase of the MRC framework. The MRC outlines a common set of core elements:  considering context, developing and refining programme theory, engaging stakeholders, identifying key uncertainties, refining the intervention and economic considerations (Skivington *et al.* 2021). These core elements should be considered early and continually taken into consideration throughout the research process, especially when moving between phases of the framework (Skivington *et al.* 2021).  Also, the MRC framework is specific to the development of complex interventions i.e. interventions which contain several interacting components such as the range of behaviours targeted, the variability of outcomes, and the degree of permitted flexibility or tailoring of the intervention itself (Skivington *et al.* 2021). As the current qualitative findings will be used to inform a future complex intervention design, we believe the MRC framework is more suitable than Sacket’s triangle to inform this complex intervention development. |
| 6 | **Methods 5. Excellent to use reporting framework (reassuring because i am not a qual researcher)** | Thank you for this comment. |
| 7 | **6. How far after stroke were you interested in? the intro suggests that views on cognitive impairment changes over time?** | As the optimum timing of cognitive rehabilitation post-stroke is still unclear (5), recruitment of people post-­­­­stroke was not specified to either inpatient, rehabilitation or community settings, and so, we did not specify a time post-stroke for participants to be eligible for inclusion. We have added this detail to the manuscript. Please see Methods, Page 8, Line 12-14. |
| 8 | **7. Excellent to make arrangements for those with communication difficulties - so important to have their voice.** | Thank you for this comment. |
| 9 | **8. not sure that TIDier is relevant here.** | The Template for Intervention Description and Replication (TIDieR) checklist TIDier was used as a tool to ensure detail description of specific intervention components, specifically, the who, what, where, when, why and how of the intervention design. Therefore, the reference to the TIDIER checklist in Methods: Data Collection, Page 10 is relevant as it informed the questions for intervention development in the interview schedule. This has been re-worded to clarify the relevance of the TIDIER in the development of the interview schedule. Please see Methods; Data Collection, Page 10, Line 13-15. |
| 10 | **Results 9. re my point 6 - it would be good to know a little more about the PpS - there can be such heterogeneity depending on stroke type, severity, age and stage.** | Thank you for this comment. This has been brought up by Reviewer 3 (Comment 11) also and so, we appreciate the need to address this. We have summarized the participant demographics in the following tables: Table 1 (demographics of people post-stroke), Table 2 (demographics of caregivers) and Table 3 (demographics of HCPs) in the results section. Please see Results, Page 13 and 14, Table 1, table 2, table 3. |
| 11 | **Discussion 9. I think there is a very clear message here - not a lot of support for single focused interventions for cognition per se but rather cognitive skills being integrated into everything they do... this has implications for training of HPs and for structuring rehab sessions. This would be easy to test - standard Rehab sessions versus standard and cognitive cues and demands added. May i suggest you discuss this?** | Thank you for this comment. Please see Discussion, Page 25, Line 18-26, and Page 26 Line 1-16, where we have discussed multicomponent interventions.  We agree that the findings of the current study may have implication for training of HCPs, however, given that the multicomponent intervention has not been tested yet, we cannot make such a recommendation as it is beyond the scope of this study. However, we have added further detail on the development of a future feasibility study, which will be informed by the current qualitative findings. Please see Discussion, Page 28, Line 19-21. |

**References:**

1.	Tatemichi T, Paik M, Bagiella E, Desmond D, Pirro M, Hanzawa L. Dementia after stroke is a predictor of long-term survival. Stroke. 1994;25(10):1915–9.

2.	Rasquin SM, Lodder J, Ponds RW, Winkens I, Jolles J, Verhey FR. Cognitive functioning after stroke: a one-year follow-up study. Dementia and geriatric cognitive disorders. 2004;18(2):138–44.

3.	Nys G, Van Zandvoort M, De Kort P, Jansen B, Kappelle L, De Haan E. Restrictions of the Mini-Mental State Examination in acute stroke. Archives of clinical neuropsychology. 2005;20(5):623–9.

4.	Malterud K, Siersma VD, Guassora AD. Sample size in qualitative interview studies: guided by information power. Qualitative health research. 2016;26(13):1753–60.

5.	McDonald MW, Black SE, Copland DA, Corbett D, Dijkhuizen RM, Farr TD, et al. Cognition in stroke rehabilitation and recovery research: Consensus-based core recommendations from the second Stroke Recovery and Rehabilitation Roundtable. International Journal of Stroke. 2019;14(8):774–82.
