# Supplementary material for: Exploring the perspectives of key stakeholders on the design and delivery of a cognitive rehabilitation intervention for people post-stroke
Source: PLoS One. 2022 Jun 16;17(6):e0269961. doi: 10.1371/journal.pone.0269961 (PMC9202836; doi:10.1371/journal.pone.0269961)
Supplement: Peer review history

# Peer review: Decision Letter 0

**DOI:** 10.1371/journal.pone.0269961.r001

**Authors:** Dylan A Mordaunt

25 Feb 2022

PONE-D-22-02317Exploring the perspectives of key stakeholders on the design and delivery of an intervention to rehabilitate cognitive deficits in people post-strokePLOS ONE

Dear Dr. O' Donoghue,

Thank you for submitting your manuscript to PLOS ONE. After careful consideration, we feel that it has merit but does not fully meet PLOS ONE’s publication criteria as it currently stands. Therefore, we invite you to submit a revised version of the manuscript that addresses the points raised during the review process.

Please submit your revised manuscript by Apr 11 2022 11:59PM. If you will need more time than this to complete your revisions, please reply to this message or contact the journal office at plosone@plos.org. When you're ready to submit your revision, log on to https://www.editorialmanager.com/pone/ and select the 'Submissions Needing Revision' folder to locate your manuscript file.

Please include the following items when submitting your revised manuscript:

- A rebuttal letter that responds to each point raised by the academic editor and reviewer(s). You should upload this letter as a separate file labeled 'Response to Reviewers'.
- A marked-up copy of your manuscript that highlights changes made to the original version. You should upload this as a separate file labeled 'Revised Manuscript with Track Changes'.
- An unmarked version of your revised paper without tracked changes. You should upload this as a separate file labeled 'Manuscript'.

If you would like to make changes to your financial disclosure, please include your updated statement in your cover letter. Guidelines for resubmitting your figure files are available below the reviewer comments at the end of this letter.

If applicable, we recommend that you deposit your laboratory protocols in protocols.io to enhance the reproducibility of your results. Protocols.io assigns your protocol its own identifier (DOI) so that it can be cited independently in the future. For instructions see: https://journals.plos.org/plosone/s/submission-guidelines#loc-laboratory-protocols. Additionally, PLOS ONE offers an option for publishing peer-reviewed Lab Protocol articles, which describe protocols hosted on protocols.io. Read more information on sharing protocols at https://plos.org/protocols?utm_medium=editorial-email&utm_source=authorletters&utm_campaign=protocols.

We look forward to receiving your revised manuscript.

Kind regards,

Dylan A Mordaunt, MB ChB, MPH, MHLM, FRACP, FAIDH

Academic Editor

PLOS ONE

Journal Requirements:

When submitting your revision, we need you to address these additional requirements.

1. Please ensure that your manuscript meets PLOS ONE's style requirements, including those for file naming. The PLOS ONE style templates can be found at

https://journals.plos.org/plosone/s/file?id=wjVg/PLOSOne_formatting_sample_main_body.pdf and

https://journals.plos.org/plosone/s/file?id=ba62/PLOSOne_formatting_sample_title_authors_affiliations.pdf

2. We note that the grant information you provided in the ‘Funding Information’ and ‘Financial Disclosure’ sections do not match.

When you resubmit, please ensure that you provide the correct grant numbers for the awards you received for your study in the ‘Funding Information’ section.

3. We note that you have stated that you will provide repository information for your data at acceptance. Should your manuscript be accepted for publication, we will hold it until you provide the relevant accession numbers or DOIs necessary to access your data. If you wish to make changes to your Data Availability statement, please describe these changes in your cover letter and we will update your Data Availability statement to reflect the information you provide.

4. Please ensure that you refer to Figure 1 in your text as, if accepted, production will need this reference to link the reader to the figure.

Additional Editor Comments:

Thank you for your submission. We received a range of feedback. One author recommended reject, however in the context of the PLoS One framework, although it is worth the authors considering his feedback with regards to novelty, I don't think these influence the criteria for submission. With regards to the criteria for publication:

1. The study presents the results of original research. Reviewer 3 has raised concerns novelty, but not originality.

2. Results reported do not appear to have been published elsewhere.

3. Experiments, statistics, and other analyses are performed to a reasonable standard, but we have generously detailed suggestions from the reviewers that should be addressed.

4. Conclusions are presented in an appropriate fashion and are supported by the data.

5. The article is presented in an intelligible fashion and is written in standard English.

6. The research meets all applicable standards for the ethics of experimentation and research integrity.

7. The article adheres to appropriate reporting guidelines and community standards for data availability.

[Note: HTML markup is below. Please do not edit.]

Reviewers' comments:

Reviewer's Responses to Questions

**Comments to the Author**

1. Is the manuscript technically sound, and do the data support the conclusions?

The manuscript must describe a technically sound piece of scientific research with data that supports the conclusions. Experiments must have been conducted rigorously, with appropriate controls, replication, and sample sizes. The conclusions must be drawn appropriately based on the data presented. 

Reviewer #1: Yes

Reviewer #2: Partly

Reviewer #3: Yes

Reviewer #4: Yes

**********

2. Has the statistical analysis been performed appropriately and rigorously? 

Reviewer #1: N/A

Reviewer #2: Yes

Reviewer #3: N/A

Reviewer #4: N/A

**********

3. Have the authors made all data underlying the findings in their manuscript fully available?

The PLOS Data policy requires authors to make all data underlying the findings described in their manuscript fully available without restriction, with rare exception (please refer to the Data Availability Statement in the manuscript PDF file). The data should be provided as part of the manuscript or its supporting information, or deposited to a public repository. For example, in addition to summary statistics, the data points behind means, medians and variance measures should be available. If there are restrictions on publicly sharing data—e.g. participant privacy or use of data from a third party—those must be specified.

Reviewer #1: Yes

Reviewer #2: Yes

Reviewer #3: Yes

Reviewer #4: Yes

**********

4. Is the manuscript presented in an intelligible fashion and written in standard English?

PLOS ONE does not copyedit accepted manuscripts, so the language in submitted articles must be clear, correct, and unambiguous. Any typographical or grammatical errors should be corrected at revision, so please note any specific errors here.

Reviewer #1: Yes

Reviewer #2: Yes

Reviewer #3: Yes

Reviewer #4: Yes

**********

5. Review Comments to the Author

Please use the space provided to explain your answers to the questions above. You may also include additional comments for the author, including concerns about dual publication, research ethics, or publication ethics. (Please upload your review as an attachment if it exceeds 20,000 characters)

Reviewer #1: Dear authors

Thank you for this manuscript, it is pertinent, interesting and largely well structured. I am making mostly minor rewording/restructuring suggestions with some requests for further detail on methods, for clarity, completeness and readability.

Abstract and 583-586

‘Evidence based and stakeholder informed’ is implicit. Suggest simply replace with 'Taken together with existing quantitative evidence, these findings will inform the development of a feasibility trial, examining patient and process outcomes, to rehabilitate cognitive deficits poststroke.'

63: “varying incidence rates between 20-80% of PpS” - clarify this is stroke of any aetiology?

65-66: “Data from a prospective population-based stroke register found the prevalence of cognitive

deficits in 22% of people at five years post-stroke and 21% of people at 14 years post-stroke (7).”

Needs rewording e.g. the prevalence of cognitive deficits at 5 years post stroke was 22%...

67: “Cognitive impairment post-stroke is shown to be independently associated”

Reword: has been shown to be independently associated OR is independently assoc...

75-76: “factors that could facilitate empowerment and enable PpS to regain control

over their everyday life” Define empowerment, or leave it out as I suspect ‘regain control over’ covers it.

79-81: This has been superseded by a Stroke Association/James Lind Alliance PSP (2021) which puts cognitive research at #2, after psychological

81-82: I would leave this sentence out here as you discuss/cite stakeholder involvement later

98-99: so did (27) only focus on a particular intervention type? That would make the distinction from present study clearer. What did (27) conclude?

96-107 I would reorganise this para to avoid repetition. “Cog rehab is defined as…” then your systematic review of 64 studies, then say the previous quali enquiry only focused on a narrow remit (if it did), hence the need for present quali study looking at wider range of interventions etc.

109-110 can you give an example of negative effects from the cited reference

115 HCPs – use acronym from the first instance of healthcare professional in main text

131 these findings – clearer to say “the findings from the present study”

Sampling and recruitment

A brief definition of Information power would be helpful.

Was the information leaflet sent at the same time as the invitation letter?

How long did participants have to consider before consent was sought?

174 capacity assessed on a continual basis – surely not for a one-off interview? Capacity wouldn’t be expected to fluctuate during the course of a single interview. How did you assess capacity?

Methods

Although you mention reflexive thematic analysis and methodological rigour several times, I don’t find this convincing as there is no evidence of reflexivity – no fieldnote excerpts. And memo-ing is not in and of itself reflexive – depends what you’re noting down. How did your biases affect data collection and analysis/interpretation? I appreciate you may not have the space to discuss in any detail.

Data analysis

I found this section repetitive…peer debriefing, continuous discussion, debriefing mentioned again in the six stages section – are these the same conversations? You mention fieldnotes/diary twice.

Findings

440: who thought interventions should be OT-led – anyone other than the OT?

250-272 This section is repetitive e.g. ‘context-specific, meaningful goals were noted to promote engagement’ is there in 2 or 3 different forms.

255 not obvious what the intervention would be, what do the HCPs do to ‘engage’ PpS?

288-289 Probably some/all were not realistically going to get back to their pre-stroke ‘normal’

296 'benefits of an interdisciplinary approach in the delivery' - correct to 'in delivering this type of intervention'

312-319 Overlap with/repetition of theme from your first section – context and meaningfulness

Discussion

535 clearer to say ‘lack of strong evidence of combined PA and cog rehab interventions’ if that’s what you mean

543-545 largely repeats first sentence in para

Reviewer #2: Thank you for the opportunity to review this paper reporting an approach to developing a multidisciplinary intervention to rehabilitate cognitive deficits in people after a stroke: a relevant topic. A qualitative study was undertaken through interviews with people post-stroke, carers and rehabilitation healthcare professionals. The findings were mixed with different views expressed on topics such as when to initiate rehabilitation. The authors identified four themes for the design and development of an intervention.

Reference 28 is cited several times in the paper. For example, on page 5, “as evidenced from a previous systematic review (28). O’ Donoghue et al. (28) conducted a systematic review of 64 studies addressing any type of non-pharmacological rehabilitation intervention which may improve cognitive function in people post-stroke.” and “Taken together with quantitative evidence from our systematic review (28)…” Reference 28 is to the protocol for a systematic review written by the first author of this paper. The same protocol paper is cited again as reference 49, “The interview guide, (supplementary material), was informed by our previous systematic review (49)…”. Based on Google Scholar and PubMed searches, the paper resulting from the protocol does not appear to have been published,. The results reported in the current paper from reference 28 are thus not in the public domain. This has implications for both the future publication of the paper based on the protocol as some of the results will have been presented in this paper if it is accepted, and the veracity of the information provided in this paper is in question if the paper, references 28 and 49, has not been published.

The sample size of 30 interviewees was guided by ‘information power’ as defined by Malterud, who in explaining the process states, “For each of these items, we have proposed dimensions along a continuum where researchers are invited to position themselves and their study to assess an approximate number of participants needed for responsible analysis. We argue that such an assessment should be stepwise revisited along the research process and not definitely decided in advance. In this way, recruitment can be brought to an end when the sample holds sufficient information power. Still, the model may offer support also in the initial planning of a qualitative interview study.” There is no mention of stepwise analysis of interview findings in the methods section. Was this done? If not, how was the sample size of 30 determined.

One of the inclusion criteria for people post-stroke was “Able to verbally communicate over the phone/ via telecommunication platform” (Line 155). In lines 179-180, it is stated that “In cases where the individual post-stroke was unable to effectively communicate in a semi structured interview setting, we informed participants that proxy respondents may be used.” If they were not able to communicate effectively, why were they included and interviewed?

The five carers interviewed were the wives of five of the people post-stroke who were interviewed. What is the effect of this on the spectrum of information that can be gathered from these five pairs? Does it lessen the ‘information power’? Granted, they will know how the stroke event has changed their partner, but what effort was made to find carers who were not partners of the patient? What additional insight may they have provided?

Methods, line 178: to what does the abbreviation PPI refer?

Limitations: the absence of physicians in the study is noted, but the reason for their exclusion, the desire “explore the management of cognitive impairment post-stroke using rehabilitation interventions with no emphasis on pharmacological interventions” appears ill-founded unless doctors are to be specifically excluded from the multidisciplinary rehabilitation process. Doctors may well prescribe medications irrespective of the rehabilitation plan, especially if they are not part of the process.

Limitations: it is noted the findings may be specific to the Republic of Ireland and may not be representative of all patients-post stroke. It should also be noted that any programme which is developed from this study will also be relevant to similar health systems with similar resources, both human and financial, but not necessarily applicable to less-resourced settings.

In Figure 1, what status was afforded to, ‘individual interests of persons must be encouraged’ and ‘variety of interventions can stimulate a real-world context’ are they subthemes or individual themes that were not chosen to be included among the four key themes and if so, why?

Data collection, lines 198-204: is the information provided about the lead author relevant to the paper?

The authors have chosen to identify those who were involved in the various aspects of the study. The lead author is identified several times. PB, SH and DL are identified with respect to coding, line 216, to peer debriefing sessions with the lead author in line 230 and PB for other actions in lines 217 and 225. What roles did the other four authors play in the study?

Reviewer #3: The manuscript is a report of a qualitative analysis of 30 interviews with stakeholders involved in delivery or receipt of cognitive rehabilitation post-stroke. It is stated that a goal of the manuscript is to inform the development of an evidence based and stakeholder informed feasibility trial. However because little detail is provided about how the results are applied to that goal the manuscript does not seem to have helped achieve that milestone.

The findings are of interest, yet it is not clear whether the findings are new or how they add to the literature, so a perspective on how the findings would influence the development of an intervention or trial would be a useful addition.

The figure illustration has arrows in the wrong place for the subtheme for theme #1.

The Methods describe that sample size is achieved with Information Power. I would suspect that this was achieved, however the authors do not provide any description for why the study met the criteria underlying the concept. A careful defense of the broad nature of the different types of interventions that would be under consideration here and the disparate people interviewed because those would tend to diminish an argument that Information Power was achieved with the moderate number of interviews.

The recruitment is not clearly described? Also, the number invited / declined to the interview was not found in the manuscript.

There is no description of the length of interviews (in a unit of time), the length of the transcripts (in pages, quotes, or words). It is thus difficult to judge whether the sample was sufficient.

The manuscript should include a box or table with a listing of acronyms used in the text and their meanings.

P17 – the author state that “majority of participants were in agreement that community-based best” but the following quote used as an example states “back to home is best” – The discrepancy needs to be resolved or explained.

There are some statements that are confusing e.g., P20 “with session length varying from 30 minutes up to one hour and 30 minute long sessions.”

There are several supplemental files provided with the manuscript, but it seemed that only one was referenced in the manuscript. (i.e. p7: “Interview scripts are available as supplemental material”).

The participant information provided in a supplementary file, may be more accessible and informative if summarized in the result section.

It is not clear what the authors intent is for the purpose of the supporting file ‘Implications for Rehabilitation’. This information should be referenced in the text or incorporated in the manuscript.

Reviewer #4: Thank you for the opportunity to review this paper on the perspectives of cognitive rehabilitation after stroke. i make the following comments and queries for your consideration:

Abstract

1. the translation of the findings into an eventual intervention is welcomed.

2. perhaps change the term to cognitive impairment rather than deficit?

Introduction

3. excellent scene setting and justification up to page 5; then the text goes specifically into memory having just justified a more global perspective in this study?

4. and the final paragraph in the intro also is a bit disjointed. Is the point that you want to produce an evidence-based intervention? then you can justify by pointing out that an evidence base includes - effectiveness evidence, expert opinion and patient values and preferences. so there is your justification. That may be what the MRC says but i am not familiar with whereas everyone knows Sackets triangle.

Methods

5. Excellent to use reporting framework (reassuring because i am not a qual researcher)

6. How far after stroke were you interested in? the intro suggests that views on cognitive impairment changes over time?

7. Excellent to make arrangements for those with communication difficulties - so important to have their voice.

8. not sure that TIDier is relevant here.

Results

9. re my point 6 - it would be good to know a little more about the PpS - there can be such heterogeneity depending on stroke type, severity, age and stage.

Discussion

9. I think there is a very clear message here - not a lot of support for single focused interventions for cognition per se but rather cognitive skills being integrated into everything they do... this has implications for training of HPs and for structuring rehab sessions. This would be easy to test - standard Rehab sessions versus standard and cognitive cues and demands added. May i suggest you discuss this?

**********

6. PLOS authors have the option to publish the peer review history of their article (what does this mean?). If published, this will include your full peer review and any attached files.

If you choose “no”, your identity will remain anonymous but your review may still be made public.

**Do you want your identity to be public for this peer review?** For information about this choice, including consent withdrawal, please see our Privacy Policy.

Reviewer #1: No

Reviewer #2: No

Reviewer #3: No

Reviewer #4: No

[NOTE: If reviewer comments were submitted as an attachment file, they will be attached to this email and accessible via the submission site. Please log into your account, locate the manuscript record, and check for the action link "View Attachments". If this link does not appear, there are no attachment files.]

While revising your submission, please upload your figure files to the Preflight Analysis and Conversion Engine (PACE) digital diagnostic tool, https://pacev2.apexcovantage.com/. PACE helps ensure that figures meet PLOS requirements. To use PACE, you must first register as a user. Registration is free. Then, login and navigate to the UPLOAD tab, where you will find detailed instructions on how to use the tool. If you encounter any issues or have any questions when using PACE, please email PLOS at figures@plos.org. Please note that Supporting Information files do not need this step.

# Author comment: Author response to Decision Letter 0

**DOI:** 10.1371/journal.pone.0269961.r002

18 May 2022

I have responded to reviewers in a separate uploaded document titled "Response to Reviewers." If responses are required in a different format, I am happy to do so.

Attachment Submitted filename: Response to reviewers.docx Click here for additional data file.

# Editor report: Decision Letter 1

**DOI:** 10.1371/journal.pone.0269961.r003

**Authors:** Dylan A Mordaunt

1 Jun 2022

Exploring the perspectives of key stakeholders on the design and delivery of a cognitive rehabilitation intervention for people post-stroke

PONE-D-22-02317R1

Dear Dr. O' Donoghue,

We’re pleased to inform you that your manuscript has been judged scientifically suitable for publication and will be formally accepted for publication once it meets all outstanding technical requirements.

Within one week, you’ll receive an e-mail detailing the required amendments. When these have been addressed, you’ll receive a formal acceptance letter and your manuscript will be scheduled for publication.

An invoice for payment will follow shortly after the formal acceptance. To ensure an efficient process, please log into Editorial Manager at http://www.editorialmanager.com/pone/, click the 'Update My Information' link at the top of the page, and double check that your user information is up-to-date. If you have any billing related questions, please contact our Author Billing department directly at authorbilling@plos.org.

If your institution or institutions have a press office, please notify them about your upcoming paper to help maximize its impact. If they’ll be preparing press materials, please inform our press team as soon as possible -- no later than 48 hours after receiving the formal acceptance. Your manuscript will remain under strict press embargo until 2 pm Eastern Time on the date of publication. For more information, please contact onepress@plos.org.

Kind regards,

Dylan A Mordaunt, MD, MPH, FRACP

Academic Editor

PLOS ONE

Additional Editor Comments (optional):

Thank you for your resubmission This now meets the criteria for publication.

Reviewers' comments:

# Editor report: Acceptance letter

**DOI:** 10.1371/journal.pone.0269961.r004

**Authors:** Dylan A Mordaunt

8 Jun 2022

PONE-D-22-02317R1 

Exploring the perspectives of key stakeholders on the design and delivery of a cognitive rehabilitation intervention for people post-stroke   

Dear Dr. O' Donoghue:

I'm pleased to inform you that your manuscript has been deemed suitable for publication in PLOS ONE. Congratulations! Your manuscript is now with our production department. 

If your institution or institutions have a press office, please let them know about your upcoming paper now to help maximize its impact. If they'll be preparing press materials, please inform our press team within the next 48 hours. Your manuscript will remain under strict press embargo until 2 pm Eastern Time on the date of publication. For more information please contact onepress@plos.org.

If we can help with anything else, please email us at plosone@plos.org. 

Thank you for submitting your work to PLOS ONE and supporting open access. 

Kind regards, 

PLOS ONE Editorial Office Staff

on behalf of

Associate Professor Dylan A Mordaunt 

Academic Editor

PLOS ONE
